# Liquid‐in‐Liquid Prints: High‐Density Biochemically Encoded Information Preserved in Microdroplet Arrays

**DOI:** 10.1002/adma.202516338

**Published:** 2025-11-24

**Authors:** Maximilian Breitfeld, Robert Strutt, Leonard Fröhlich, Claudius L. Dietsche, Sebastian Bargfrede, Petra S. Dittrich

**Affiliations:** ^1^ Department of Biosystems Science and Engineering ETH Zürich Schanzenstrasse 44 Basel 4056 Switzerland

**Keywords:** automation, chemical information, droplet microfluidics, encoding, high‐throughput assays, liquid state printing

## Abstract

Liquids are dense repositories of information, challenged only by how well their compositions are defined, preserved, accessed, or measured. The precise spatial patterning of solutes within a bulk liquid is challenging since diffusion disperses local concentrations and thereby attenuates functionality. Herein, a new concept is introduced for writing and preserving information in the liquid state through liquid‐in‐liquid microdroplet array printing. This technology produces fine resolution, 2D liquid structures, composite of indexed water‐in‐oil droplet pixels each with a precise composition, a high spatial resolution and a tight inter‐pixel pitch. With extreme control over droplet composition and by applying standard and custom encoding schemes, various forms of information are written biochemically such as images, QR codes, text characters and words. As a composite material, reversible phase transitions between dissolved liquid and crystallized solid states control information encryption and decryption. Compared to current liquid printing and chemical encoding paradigms, ours introduces a fundamentally new precedent for deterministically programming information release, exchange or decay without stimuli or physical processing. Further computational principles such as error correction and information storage are demonstrated. These micro‐liquid patterns are relevant to any application based on precise liquid handling such as information theory, materials design and biological assays.

## Graphical abstract

This work introduces a novel approach for encoding and storing information in the liquid state in microdroplet arrays. These liquid‐in‐liquid prints are generated by a droplet printing system capable of dynamically setting the composition of each droplet pixel. Information in the form of color images, QR codes, text characters, words and basic programmes are biochemically written into micro‐liquid patterns.

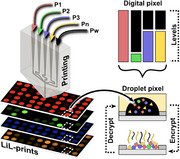

## 1 Introduction

Precise control over composition, spatial location and timing is universal to the metering and delivery of liquids in the natural sciences. Depositing in the liquid and drying into a solidified state underpins inkjet,^[^ ^1^, ^2^ ^]^ hydrogel,^[^ ^3^ ^]^ and 3D printing, which have been refined for the fabrication of flexible sensors,^[^ ^4^ ^]^ structural color surfaces,^[^ ^5^, ^6^, ^7^ ^]^ solar cells^[^ ^8^ ^]^ and materials for data security.^[^ ^9^, ^10^ ^]^ Such automated modes of liquid handling effectively translate digital information into liquid patterns that determine material compositions. Chemical encoding has been proposed for long term data storage, data security and transport since electriconic and magnetic storage degrade on the order of tens of years.^[^ ^11^, ^12^, ^13^, ^14^, ^15^, ^16^ ^]^ In such applications, recovering information relies on the maintenance and analysis of a chemical compositional identity which is often dried in the solid state. Preserving the liquid state on the order of tens of hours is essential in the life sciences for investigating interactions between cells and their microenvironments.^[^ ^17^, ^18^ ^]^ Liquids are typically deposited by pipette‐based handling into indexed wells which are delineated and maintained by physical barriers. This separation prevents compositional mixing such that information, which is either fixed (chemical encoding) or dynamic (biochemical assays), remains spatially defined and therefore interpretable. Multiwell plates are limited in sample density, sustainability and application in many fields.^[^ ^19^ ^]^ Fundamentally divergent approaches are required to further miniaturize liquid printing and patterning paradigms.

Droplet microfluidics has well‐addressed the need for creating small liquid volumes, enabling continuous, high‐speed formation of nano‐ and pico‐liter reactors.^[^ ^20^, ^21^ ^]^ When sustained in an otherwise immiscible medium, droplets are increasingly conceptualized as building blocks for liquid devices and materials.^[^ ^22^, ^23^, ^24^, ^25^ ^]^ Structural resolution depends on the production and size of droplets and crucially how well they are preserved. Most droplet microfluidic technologies cannot spatially organize and maintain low volume droplets with a per‐droplet compositional identity in an appreciable, industrially relevant timescale. In this respect, a microdroplet array consists of droplets adhered on a surface and typically covered by oil to prevent evaporation.^[^ ^26^, ^27^, ^28^, ^29^, ^30^ ^]^ The array is open and accessible from above, uniquely facilitating manipulation and indexability. Previously, we introduced a liquid printing process without thermal and under minimal mechanical agitation for rapid biochemical printing of large microdroplet arrays, however only with simple composition gradients.^[^ ^18^ ^]^

Inspired by solid state material fabrication with inkjet and related 3D printing techniques,^[^ ^17^, ^31^ ^]^ we have built a miniaturized liquid droplet printer that produces nanoliter volumes with precise compositions in microdroplet arrays. We refer to the printed material as a liquid‐in‐liquid (LiL) print. Differing from current techniques for printing liquid state materials,^[^ ^22^, ^32^, ^33^, ^34^ ^]^ here, droplet compositions comprise of solutes and suspensions printed on a spatially resolved, per pixel basis in a single print run by defining the pressure applied to component lines in a printhead. To evidence the potential breadth of this printing technology, we print droplets comprised of dyes, drugs, media and cells in distinct concentrations such that each droplet contains discretized information in the form of a compositional identity. Readout relies on fluorescence to reveal information encoded globally at the macro scale of the LiL print. With these materials, we render numerous computation principles including standard and custom encoding schemes, error correction, encryption, decryption, storage and programmed information release and decay in the liquid state. LiL prints thereby miniaturize multiwell plate experimentation as relevant to chemical encoding and the spatiotemporal programming of biochemical reactions.

## 2 Results

### 2.1 A Custom Pipeline for Printing Spatially Organized Biochemically Complex Droplet Pixels

**Figure 1:**
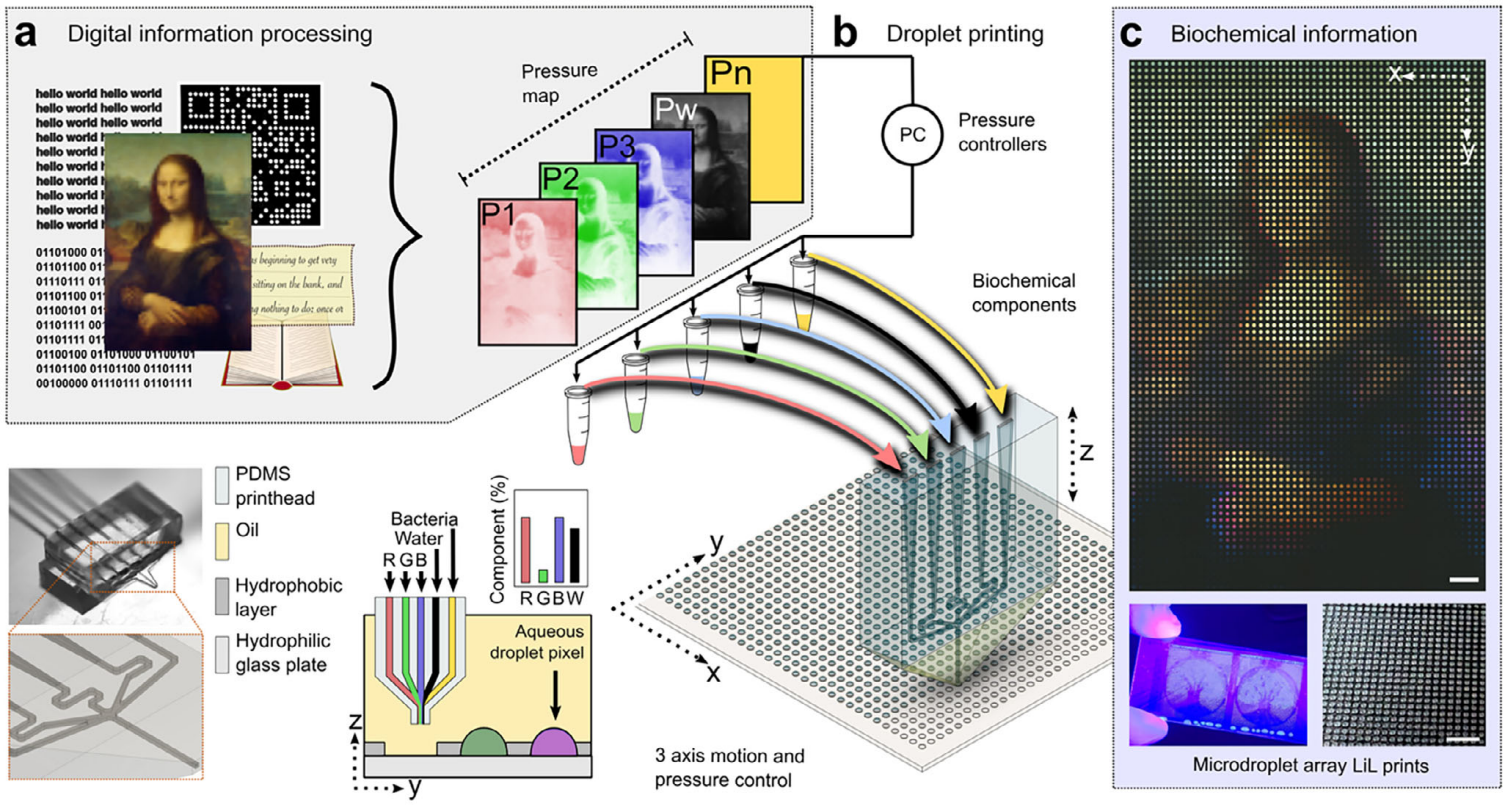
Pipeline for biochemically printing digital information in liquid‐in‐liquid (LiL) prints: a microdroplet array technology. a) Schematic showing the conversion of different information representations to biochemical droplet composition, here expanded for image printing. b) Droplets in oil are formed on a wettability patterned surface using a 5‐channel pressure‐controlled PDMS printhead. The composition is set at the point of droplet generation by the pressure ratio of up to 5 component channels (P1–Pn) in the printhead, here shown as a composite droplet color. c) From top to bottom, an example of biochemical patterning resolution in a LiL print reconstruction of the Mona Lisa by Leonardo Da Vinci (scale bar = 1 mm). LiL prints occupy the dimensions of a glass slide, right shows surface droplet morphology (scale bar = 1 mm). Printed via microdroplet arrays by stream shearing (MASS) on a 56‐dpi surface. Here, the image color is represented with differentiable fluorescent dyes accordingly, P1‐Red–Dextran Alexa Fluor 647, P2‐Green – fluorescein and P3‐Blue–Dextran Alexa Fluor 405, P4–Black–DI water. Mona Lisa by Leonardo da Vinci, (≈1503–1506; public domain image via Wikimedia Commons, courtesy of the Louvre Museum) was digitally processed and experimentally reproduced in a microdroplet array. The face of Mona Lisa was further converted into a binary pattern and reproduced in a microdroplet array.

To evidence the LiL print concept, we first aimed to translate digital image color to droplet biochemical composition. First, a digital image was processed to match the resolution of the printing surface. To access relative pixel intensities, the image was split into the levels of a core additive color model (red, green and blue). A composite pressure map was then constructed in which each level comprised a layer of the map with each then assigned to a biochemical printing component (**Figure** **1**a).

LiL prints were produced by an on‐demand, droplet‐by‐droplet printing system (Figure 1b; Figure S1, Supporting Information). This comprised of two core fabricated parts; the printing surface and a custom polydimethylsiloxane (PDMS) microfluidic device, referred to as a printhead. The printing surface was fabricated with periodic, defined hydrophilic spots with a continuous hydrophobic region around them.^[^ ^19^, ^27^, ^28^, ^35^ ^]^ The spot dimensions (250 µm spot diameter and 450 µm pitch) of the printing surface defined the image resolution. The hydrophobic region of the printing surface is analogous to an inter‐pixel spacing or a non‐emissive gap in a standard display. The printing surface was immersed in an oil (hydrofluoroether (HFE)) bath with the printhead offset above it in z, but below the bath oil–air interface. Up to five components were individually connected to pressurized sample vessels and fed via tubing into the printhead (Table S1, Supporting Infomation). The number of pressure map layers corresponded to the number of printed droplet components including a volume‐regulating buffer channel (black inlet in printhead cartoons throughout). Printed aqueous components converged into a co‐stream in a 50 × 50 × 700 µm channel (w × h × l) at the point of ejection from the printhead (Figure S2, Supporting Information). By controlling the pressure of each inlet with distinct computer programmable pressure controllers, this set the flow rate ratio in the printed stream. The droplet composition could thereby be defined at the point of droplet generation. Printed droplets which compose LiL prints are referred throughout as droplet “pixels”. A custom software orchestrated the movement of the printing surface in x and y, the movement of the printhead in z and the temporal activation of the pressure controllers according to the pressure map. Printed droplet pixels of ≈3 nL were localized through wetting to the hydrophilic spots on the printing surface (Figure S2, Supporting Information).^[^ ^18^ ^]^ Color in LiL prints was represented through concentrations of analytically resolvable droplet components. In the example in Figure 1c, the composite color is shown via concentration of three dyes with negligible cross fluorescence. When viewed by eye, the droplets appeared uniform, and optically indistinguishable (Figure 1c). When the droplet compositions were revealed through an analytical method, in this case, a fluorescence microscope, LiL prints exquisitely captured the photorealistic form, configuration and color of the original digital image.

### 2.2 Different Modes of Droplet Printing Define Print Time and Accuracy

Through control over the printing machinery, we devised different print modes (Figure S3, Supporting Information).^[^ ^18^ ^]^ One print mode relied on a continuous stream with on‐the‐fly changes to the pressure according to the relative movement of the printhead over the printing surface. We denote this mode as microdroplet arrays by stream shearing (MASS). The other was a stopped flow print mode, denoted stop‐on‐spots (SOS). This technique is based on spatially determined deposition of a fixed droplet composition with a swift motion of the printhead to detach the droplet upon spot wetting. Droplet deposition could be performed at indexed positions and in selected patterns (Figure S4, Supporting Information). Both printing methods enabled chemical and biological components to be printed without significant mechanical or thermal disturbance. Printing was less sensitive to viscosity compared to inkjet printing,^[^ ^17^, ^36^ ^]^ and we could reliably print up to ≈23 cP solutions with a single capillary.^[^ ^18^ ^]^ More details can be found in the methods, Videos S1 and S2 (print modes) and supporting Information.

**Figure 2:**
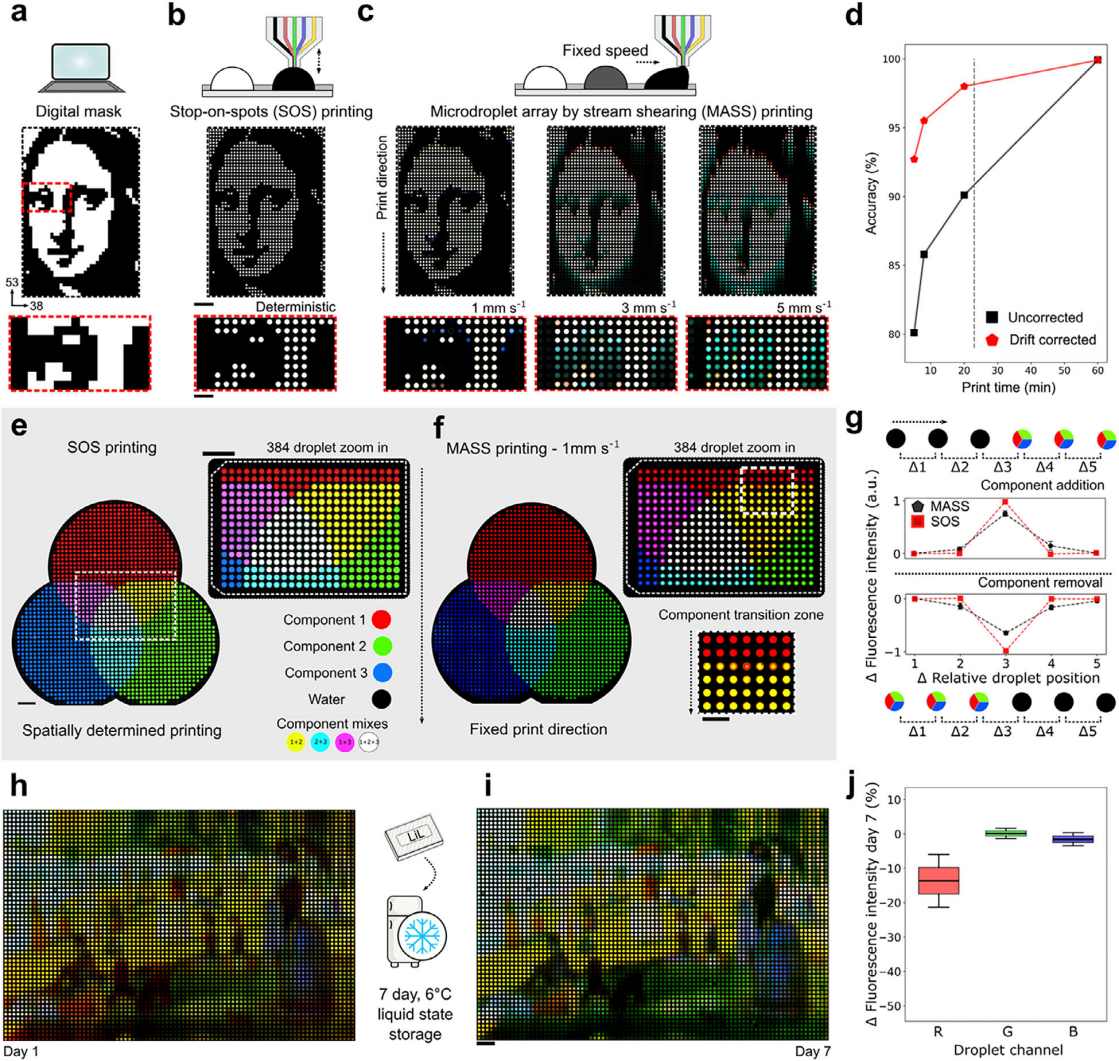
LiL print production modes and characterization. a) An example digital mask (38x53) for testing print mode accuracy with binary compositional switches. Zoom in shows an 8x15 droplet region with extreme pixel spatial variance. b) Printing the mask via SOS printing. c) Printing the mask via MASS with different surface translation speeds. In b‐c the main micrographs show the LiL print (scale bar = 2 mm) and insert shows the zoom in region (scale bar = 1 mm). d) Print mode accuracy according to data in b‐c (droplets analyzed, *n* = >1891 per point). e) A three component color wheel test motif for multi‐compositional switches printed via SOS printing (scale bar = 2 mm). Additive primary colors (RGB) indicate single biochemical compositions; overlaps indicate the presence of multiple biochemical components. Insert shows an example 24^*^16 droplet region with high pixel spatial and compositional variance (scale bar = 2 mm). f) The same motif and insert region as in e, printed via MASS with optimized settings. Additional insert shows a transition zone in the direction of the print (scale bar = 1 mm). g) Compositional accuracy at transition zones between switches for component addition or removal. Here shown for all transition zones (droplets analyzed, *n* = 654, addition, *n* = 252, removal, *n* = 402). h) Long‐term stability and performance of LiL prints shown with a print reconstruction based on “A Sunday Afternoon on the Island of La Grande Jatte” by Georges Seurat (1884–1886; public domain image via Wikimedia Commons, courtesy of the Art Institute of Chicago, digitally processed and experimentally reproduced in a microdroplet array.). Here color was represented as R–sulforhodamine B (SRB), G–Dextran Alexa Fluor 488, B–Dextran Alexa Fluor 647. I) The same print, imaged after 7 days stored in a fridge. Scale bar = 2 mm. j) Quantitative analysis of pixels between initial and stored prints (*n* = 229 droplets).

To investigate print mode accuracy, we used a canonical digital black and white image (**Figure** **2**a). Droplet pixels were colored according to the sum pressure distributed across all pressure channels (white) or the pressure predominantly applied only to the water channel (black). SOS printing was optically identical to the digital mask (Figure 2b) at the macroscale (main) and microscale (insert). Compared to SOS printing, MASS printing with a printhead movement speed of 1 mm s^−1^, or a droplet generation of ≈2 Hz, resulted in a macroscale image near identical to the mask. Faster droplet generation modes resulted in an increased compositional drift between droplets along the print direction. We quantified print mode accuracy according to the droplet compositional variance and their relative spatial distribution via a mean squared error calculation (Figure 2d; Note S1, Supporting Information). The analysis quantified the positional drift present only in MASS printing which, by eye, is accounted for due to the familiarity of the image (Figure 1c). Factoring in this shift, optimized MASS printing resulted in a 98% printing accuracy with a comparative SOS accuracy of 99.9% (Figure 2d). A binary classification of printing precision yielded comparative 100% SOS precision and 96.8% for MASS, respectively.

Next, we printed all possible component combinations through a “color wheel” motif. Analysis of the component distribution via MASS evidenced concentration variance at the wheel edges, corresponding to a composition shift during printing (Figure S5; zoom in Figure 2e,f). This was shown by a sigmoidal intensity distribution in single channels (Figure S6, Supporting Infomation). This accuracy loss was uniformly distributed for both addition and removal of a new component over two central droplets in a six‐droplet transition zone (Figure 2g). Printed solute components had a molecular weight (MW) range of 332 (green) to 10 000 (red and blue) Da. We evidenced no MW dependency on printing transition zones (Figure S7, Supporting Infomation). Comparatively, SOS printing returned a step function in component distributions in both spatial dimensions of the print. Print modes exhibited a clear tradeoff between print time and accuracy owing to the limits of the employed pressure controllers. Nonetheless, both modes were capable of extreme biochemical compositional and spatial accuracy and precision (Tables S2 and S3, Supporting Infomation). The print mode speed‐accuracy tradeoff is in relation to the employed equipment, and part fabrication. This is relevant to potential applications of LiL prints. Due to the control over the printing components, our printer can uniquely switch between deposition modes. For example, a faster print time with MASS may be more desirable where components rapidly react upon mixing. SOS on the other hand enables increased accuracy and positional indexability thereby ideal for observing large variation under subtle shifts in biochemical components.

### 2.3 Long Term Performance of Droplet Surfaces

Further, we investigated the long‐term liquid state stability of LiL prints from the perspective of the droplet morphology and the chemical partition dynamics across the liquid pattern. To test long‐term performance, we printed a LiL print with a broad color structure for by eye comparison (Figure 2h). We sealed the print within a chamber with integrated water baths to provide a closed, humid system for long term liquid droplet storage. Here, droplet morphology may have been affected by shrinkage, expansion or coalescence and the composition may have been affected by temperature, oxidation, cross reaction, photobleaching or component leakage into the oil. Broadly, compositional changes would result in signal degradation. After a week in a fridge, the print was reimaged. Remarkably, over this period we observed only one instance of droplet coalescence due to debris, and every pixel remained pinned in place. As observed by the morphology of the image, the chemical accuracy was also qualitatively maintained (Figure 2i). Pixel quantification revealed a slight decrease in fluorescence for SRB, with a negligible fluorescence shift for dextran Alexa Fluor 488 and dextran Alexa Fluor 647 (Figure 2j). Finally, all component fluorescent levels remained low in empty droplets, indicating negligible cross contamination. The slight differences are attributed due to background variation (Figure S8, Supporting Infomation). This potential for long‐term storage is applicable in many biological reaction and cultivation contexts and may be further optimized for even longer micro‐liquid pattern preservation.

### 2.4 Encoding Distinct Information in Each Droplet Component

**Figure 3:**
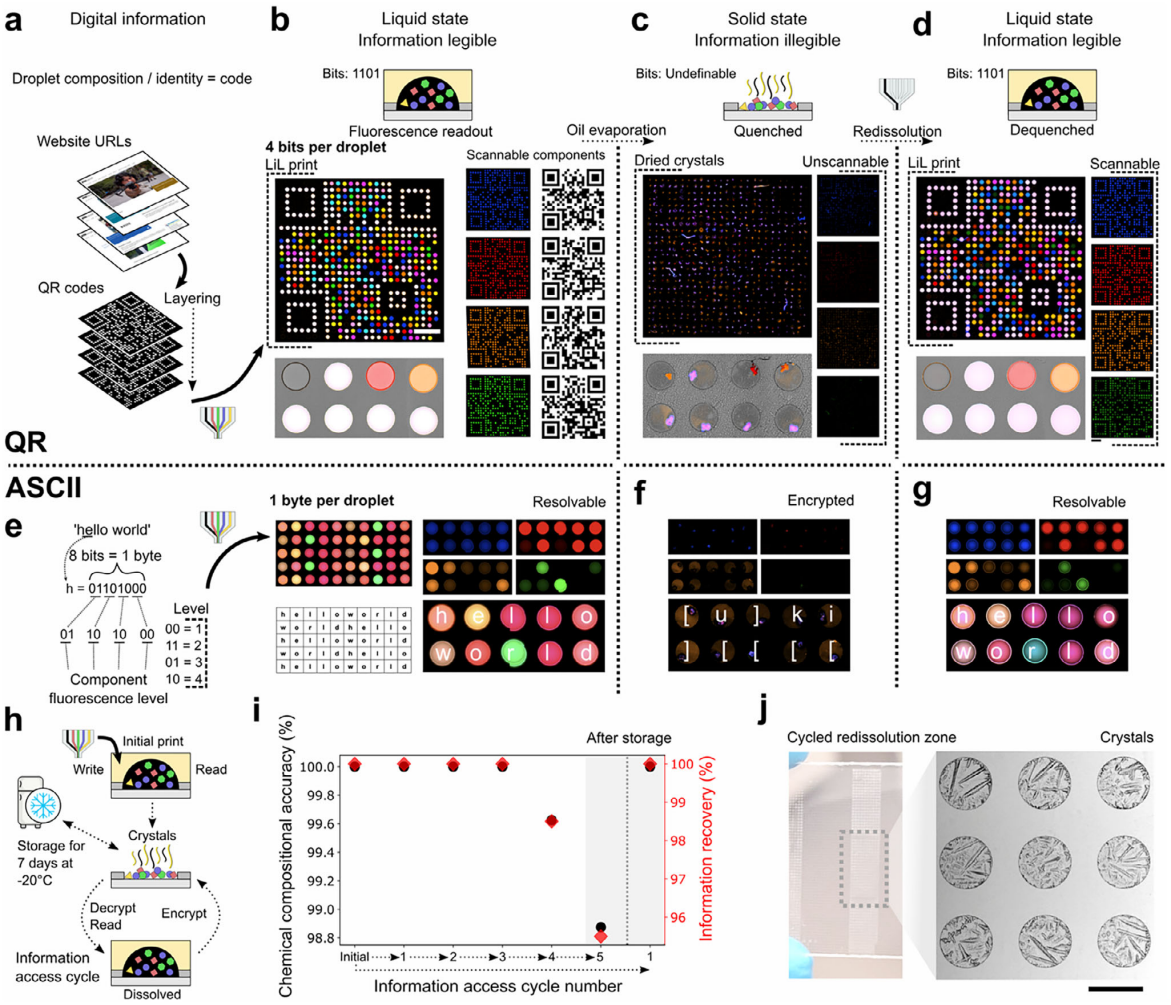
Computer readable information forms encoded into liquids with encryption through reversible phase transitions. a) Pipeline to convert QR codes or ASCII characters into droplet solute composition. b) Liquid, multi‐layer compressed QR codes in a 625‐droplet pixel code block (25 × 25 pixels). Cartoons represent the droplet solute composition as the ratio of colored symbols. Color variance in the micrographs corresponds to the droplet composition and therefore the bit identity. Information is encoded within each resolvable component. c) Encrypting information by transitioning components from dissolved liquid to localized solid crystals by evaporation. d) Redissolving the crystals and thereby decrypting the information through precise printing of water. e) Liquid byte encoding through printed component concentration level in a 10 × 5 droplet code block, eight bits per droplet. The information is determined from all components. f) Byte encryption through crystallization. g) Byte information recovery through dissolution. h) Cartoon depicting cycles of information accessibility between drying and redissolving droplet composition. Dried crystals can be placed in a freezer for long‐term storage. i) Cycled information recovery and chemical printing precision. For an initial print, cycled four times and then stored for 7 days and redissolved (*n*= 200 droplets assessed) and for an initial print stored for 7 days and then dissolved (*n* = 119). j) Photographs of crystals dried onto the printing surface within hydrophilic spots, imaged at cycle five, scale bar = 250 µm. For all, components 1–4 are in channels DAPI, Cy5, mCherry and GFP respectively. All fluorescence images are presented with the same color and brightness processing. In b–g, the main micrograph shows the 4‐channel fluorescence composite of the LiL print. In b‐d, the main micrograph scale bar = 1.8 mm. Side inserts show fluorescence micrographs in each component channel. Below the main micrograph, a brightfield and fluorescence composite micrograph show the same pixel region after each processing step, scale bar = 250 µm. In e–g the upper micrographs show each component level.

**Figure 4:**
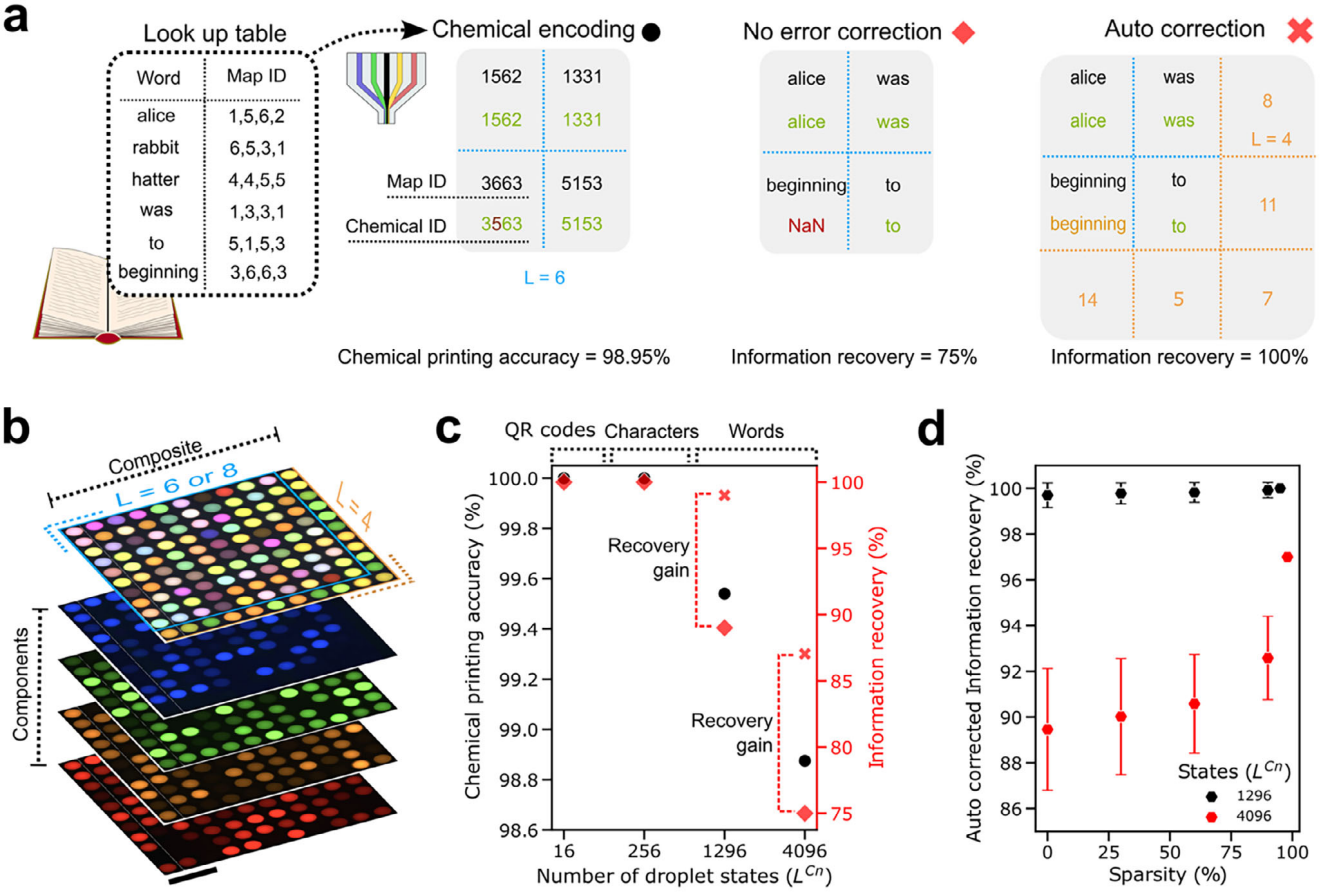
Custom encoding schemes for high information density. a) Pipeline to convert whole texts into high density droplet code blocks. Example shows error correction in chemical printing processed with and without an autonomous error correction protocol using checksum droplets. b) Micrograph of a high information density 10 × 10 liquid droplet code block (*L* = 8) with an error correction border (*L* = 4). Scale bar = 1 mm. A text section from Alice's Adventures in Wonderland (Lewis Carroll, 1865; public domain) was encoded in this microdroplet array. c) Assessment of information recovery as a function of definable droplet states with or without error correction for a canonical text (Component number (Cn) is fixed = 4, *L* = 2, 4, 6, or 8, sparsity fixed at 35%). d) Assessment of encoding sparsity on information recovery using a library of English words. Data shows mean and standard deviation of 50 algorithmic processing runs per condition. The maximal sparsity exhibited a single solution to the algorithm.

Using our printing protocols, we next translated machine readable information into LiL prints. In an information context, compared to the same relative solid state volume, solvation increases freely accessible information dimensions. Appropriately, we adapted canonical code representations with varying state densities (**Figure** **3**a; printing shown in Video S1, Supporting Infomation) via the following formula:

(1) $$\mathrm{Number}\;\mathrm{of}\;\mathrm{distinct}\;\mathrm{droplet}\;\mathrm{compositions}=L^{\mathrm{Cn}}$$

where L is the number of concentration levels printed per droplet pixel, assumed constant for each component (e.g., bits‐0 or 1, *L* = 2) and Cn is the number of differentiable pixel components or the chemical dimensions of the information (fixed as 4 in Figures 3 and 4). In each case, information was distributed across the spatial and chemical print dimensions.

We first explored 4‐bit information in each droplet pixel through quick response (QR) codes encoded in each component (Figure S9, Supporting Information). The droplet composition was analogous to a 4‐bit string, with a pattern ranging between 0000 and 1111, indicating a black or white composite pixel color respectively. The composite 2500‐bit print (11 × 11 mm) is shown in Figure 3b. As expected, the composite QR code contained no legible information and could not be scanned. Individually resolving each of the four components revealed a distinct, scannable QR code. Importantly, information could be extracted without algorithmic processing of the fluorescent signal, thresholding, correction factors or decryption. The large intensity difference between 1‐ and 0 bits rendered the information legible across a range of contrast and brightness modifications. Each QR code could be scanned directly from the screen of the microscope, for example with a mobile phone. In essence, this demonstrates the compression of multiple QR codes into the comparative space occupied typically by one, solid state QR code.

Owing to the indexability of the print, droplet pixels could be reversibly transitioned between liquid and solid states (Video S1, Supporting Infomation). We developed this as a mechanism for information encryption. In these experiments, the oil initially prevented droplet evaporation. By evaporating both the oil and, subsequently, the droplets, solutes dried in the hydrophilic spots, co‐crystallizing together (Video S1, Supporting Infomation).^[^ ^37^ ^]^ The 0‐bit contained a low concentration of the component. Co‐crystallization led to fluorescence quenching in both the 1‐ and 0‐bit droplets rendering each of the QR codes illegible (Figure 3c). The information was thereby encrypted if analyzed with the method able to otherwise decode the liquid state, despite some debris and fibers that adhered onto the surface. We added the dried print back into an oil bath and SOS printed de‐ionized (DI) water to dissolve the crystals in each spot. The redissolved surface had identical information legibility compared to the initial print (Figure 3d), demonstrating a cycle of information legibility to illegibility and back again.

In addition to 4‐bit strings, we extended the information density per pixel to a byte through resolvable concentration levels (*L* = 4, Figure 3e). The droplet composition could thereby be assigned an ASCII character barcode for each droplet pixel. We printed horizontally and vertically legible code blocks of a 10‐byte example canonical message. To decipher, we compared intensity distributions across 2 droplet code blocks (Figure S10, Supporting Infomation). Due to distinct separations in the distribution of intensities and a clear periodicity in each channel, simple thresholding could assign each identity. A confusion matrix identified 0 errors between the byte string assigned by the analysis and the digital code string. A liquid to solid phase transition quenched the intensities and led to illegible information (Figure 3f). As before, legible information could be recovered by redissolving the crystals (Figure 3g).

We investigated information accessibility robustness with an optimized redissolution protocol (Figure 3h). A 10 × 20 droplet “helloworld” code block was cycled multiple times between crystal and dissolved pixel states. The decoded byte string was proportional to the chemical compositional accuracy, and the composite information in the form of encoded characters was assessed at each stage. Remarkably, no changes were observed for the first 3 cycles, retaining total information recovery (Figure 3i). The 4^th^ cycle introduced three erroneous droplets. For the 5^th^ cycle, the crystallized pixel surface was stored in a freezer for 7 days (Figure 3j). After this period, the pixels were again redissolved and assessed, uncovering 6 more erroneous droplets. The error spatial distribution and the brightfield images (Figure S11, Supporting Infomation) indicated that fibers and debris had accumulated during multiple redissolution steps of the assessed pixel zone, causing slight compositional mixing. Another pixel zone was also investigated which had not been redissolved since the first crystallization from the initial print. After the same long‐term storage, this droplet zone gave error‐free information recovery (Figure 3i). Information decay was thereby driven by the laboratory environment and the number of access cycles, rather than intrinsic chemical decay. In short, this extends the potential of the material for storage of chemical information in either solid or liquid states.

### 2.5 Custom Encoding and Error Correction

Having successfully translated standard encoding schemes, we next designed a custom, high density encoding scheme for words (*L* = 6 or 8) (**Figure** **4**a). A canonical text was selected (the first 100 words of “Alice's Adventures in Wonderland” by Lewis Carroll), and all unique words were extracted and assigned a droplet composition identity. This gave a 35% encoding sparsity. Assuming each character as a byte, the information density was 12.6 kB inch^−1^. We designed an error correction protocol with error correction checksum droplets encoding the sum of the word characters row‐wise, column‐wise and diagonally (Figure 4b). As such, errors in the encoding droplets could be autonomously corrected by completion of the Sudoku‐like checksum scheme via look up tables to match composition to words. The print had a code rate of 0.826. We investigated droplet compositional printing accuracy and information recovery properties with or without error correction.

An increase in L resulted in only a slightly reduced compositional printing accuracy which, without error correction led to an apparent decrease in word information recovery (Figure 4c). For *L* = 6, erroneously encoding droplets comprised of only ±1 errors were randomly distributed in one component (Figure S12, Supporting Infomation). A chemical printing accuracy of >99.5% was sufficient for immediate 89% information recovery. Through autonomous error correction, 99% of the information was recoverable with a remaining false positive which could easily be solved through human interpretation of the solution, yielding 100% information recovery. For the higher encoding state (*L* = 8), a compositional accuracy of >98.8% resulted in relatively weaker information recovery with or without error correction. This was driven through introduction of −2 shifted errors and ±1 errors in up to two components per erroneous droplet, likely introduced by intrinsic fluctuations in the employed pressure controllers. These error forms significantly extended the number of solutions to the checksum correction rendering an autonomous solution with reduced legibility. The code sparsity was adjusted by producing a look up table with the 100 canonical encoding words, augmented with a random distribution of English words. Decreasing the sparsity resulted in a decay in information recovery for the *L* = 8 but not for the *L* = 6 encoding scheme (Figure 4d). Interestingly, the lexicon of the encoded text also dictated the information recovery which varied from 90.5% to 87% for the English and canonical encoding schemes, respectively, at a comparative sparsity (Figure S13, Supporting Infomation). Regardless, the distribution of errors in the prints dictated the relative capacity for information recovery. Through human grammatical interpretation, ≈95% correct information could still be recovered for all non −2 error containing droplets. These introduced encoding schemes across resolvable chemical and printed spatial dimensions thereby have the potential for high information security and density properties.

### 2.6 Deterministic Information Dynamics According to Reaction Patterning

**Figure 5:**
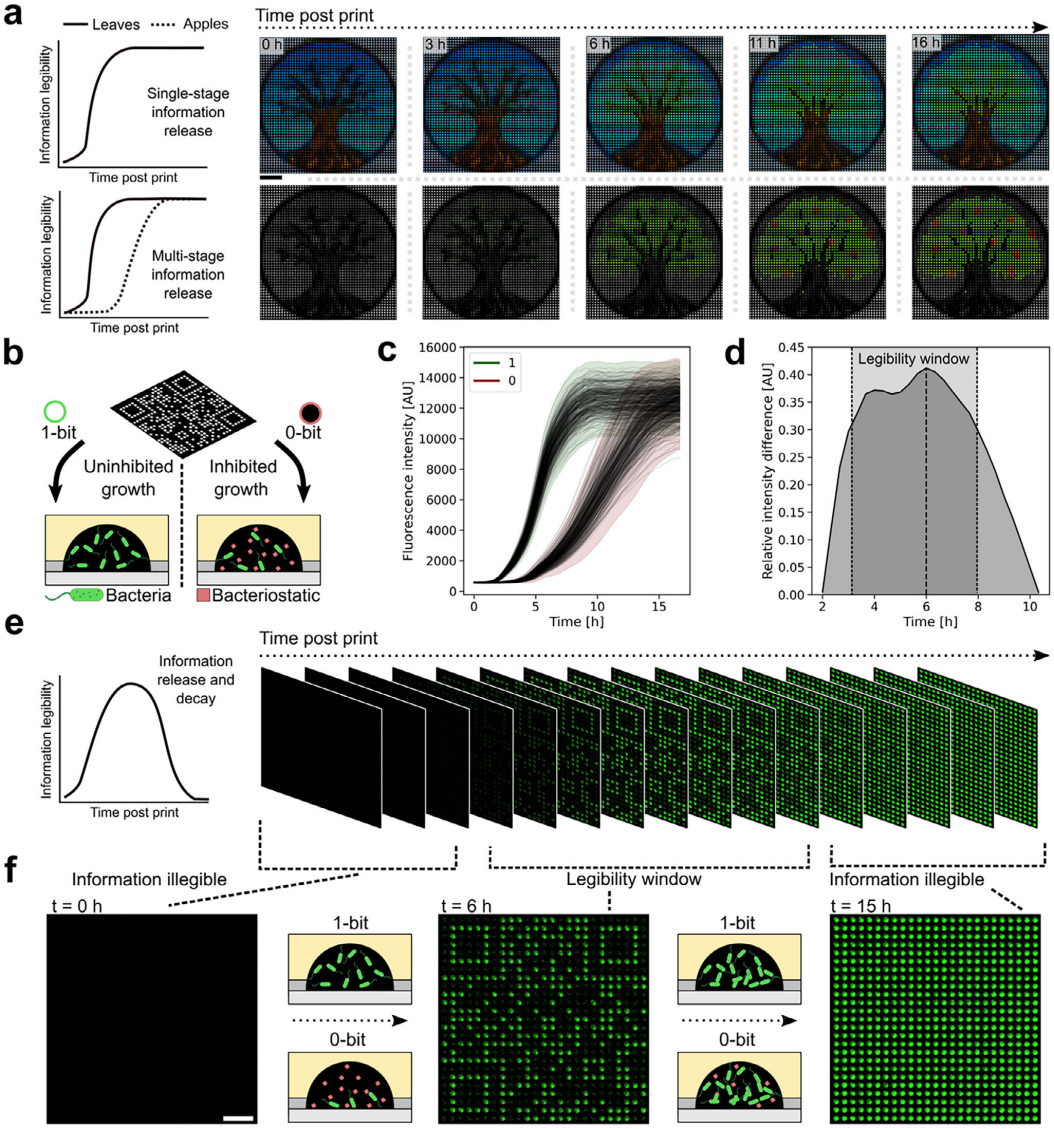
Living droplet pixels for hardcoded, deterministic control of information legibility. a) LiL prints patterned with biological cells enable passive, multi‐stage information release. Scale bar = 4 mm. b) Schematic of a 25 × 25 QR code comprised of two compositions differing in temporal trajectories and assigned to white (1‐bit) or black (0‐bit) digital QR code pixels. c) Growth profiles for each droplet in each composition, *n* = 309 and n = 316 droplets for 1‐ and 0‐ bit compositions respectively, color shade represents the confidence interval. Confidence interval defined as 3^*^standard deviation (SD). d) A plot of the difference between the lower boundary of the 1‐bit confidence interval and the upper boundary of the confidence interval of the 0‐bit. e) Fluorescence micrograph images of a QR LiL print through time in the GFP channel. f) Highlighting information legibility in the QR LiL print. Example fluorescence micrographs at key points in the temporal trajectory. Scale bar = 2 mm.

By printing non‐equilibrium solutions into droplet pixels, we expanded our concept beyond information writing and accessibility to information programmability. We engineered passive information modulation in multiple stages by printing fluorescent protein expressing bacteria and their microenvironments (**Figure** **5**a). Here, information dynamics were based on reverse engineering the bacterial growth of each droplet pixel. A dormant tree of life motif was first drawn by hand and scanned into a computer (Figure S14, Supporting Infomation). As before, the image was translated into a LiL print and through reverse‐engineering bacterial growth dynamics programmed to passively evolve over a 16 h period from winter through to spring (single stage) or through spring and summer seasons (multi‐stage). From the point of completing the print, information release was thereby deterministically set without external stimuli or interaction.

We next programmed information release and destruction in the same LiL print. We printed a QR code using bacterial cultures where one composition represented a control experiment in the absence of antibiotic (1‐bit, digitally white), and one composition represented an experiment with the same strain under a sub minimal inhibitory concentration of a bacteriostatic (chloramphenicol, 0‐bit, digitally black). Growth in the 1‐bit pixels followed a typical sigmoidal distribution (Figure 5c). A lag phase of ≈1.5 h was followed by an exponential phase that reached a plateau intensity ≈8 h post print. Comparatively, the bacteriostatic in the 0‐bit pixels resulted in an extended lag phase of ≈4 h before a relatively protracted exponential growth phase plateauing at a comparative fluorescence intensity ≈15 h. The print captured a robust dataset between control and experimental conditions, with no growth profiles outside of 3^*^SD in either population. All pixels started with the same initial bacterial OD and so the embedded QR code was initially illegible. Figure 5d shows the legibility window, a period between 3.3–8 h in the temporal trajectory during which 1‐ and 0‐bit pixels could be optically and thereby statistically distinguished. The width of the legibility window corresponded with the difference in growth profiles due to the efficacy of the antibiotic. During the legibility window, the LiL print encoded a URL to the homepage of the Federal Institute of Technology Zürich (ETH Zürich) (Figure 5e). As the 0‐bit droplet pixels grew, the QR code became increasingly illegible and from 14 h could not be scanned (Figure 5f), even with additional algorithmic processing. Moreover, when shining a UV lamp on the microarray, the QR code was scannable with a phone camera, external from a microscope. Compared to typical solid‐state materials, this temporal programming of information legibility is facile in the liquid state and here required no external stimuli or interaction (Video S2, Supporting Infomation). As such, LiL prints represent a unique approach for information encryption strategies which can leverage any responsive biochemical components printed initially out of thermodynamic equilibrium.

## 3 Discussion

We present a production method and framework for biochemically embedding information into liquids. The power of LiL prints is emergent from advances in the printing, miniaturization, resolution, indexability and control over the preservation of liquid state patterning where compositional exchange and thus information loss through diffusion is prevented. Our custom liquid printing device miniaturizes all functionalities of a standard microwell plate liquid handler, autonomously printing liquid structures with extreme spatial and compositional accuracy. Printing is solute‐agnostic and operates without thermal and under minimal mechanical agitation. Printing occurs in a bath solution with a comparative viscosity to the printed aqueous phase and importantly, without the requirement of gelation or surfactants. We demonstrate >98.8% compositional printing accuracy of up to 4096 resolvable combinations of 4 liquid samples from small stock volumes (>5 µL). In principle, numerous life science applications can benefit from this technology where highly resolved patterns of liquids and biochemicals are needed for, e.g., materials design,^[^ ^22^ ^]^ bioprinting,^[^ ^38^ ^]^ high throughput screening,^[^ ^18^, ^39^ ^]^ artificial cell production,^[^ ^30^, ^38^, ^40^ ^]^ chemical computing^[^ ^41^ ^]^ and information theory.^[^ ^42^ ^]^

Our chemical encoding demonstrates a step change when benchmarked against similar efforts in the literature (Tables S2–S6, Supporting Information). Compared to miniaturized artworks using commercial liquid handling technologies, we showcase full color, diverse samples and resolution preservation.^[^ ^43^ ^]^ Inkjet‐printed, solid state fluorescent encryption strategies are typically defined by a single bit per resolvable pixel with indexability maintained by a covalent anchoring strategy.^[^ ^11^, ^44^ ^]^ To the best of our knowledge, only 4 QR bits per pixel or 16‐pixel identities have been previously demonstrated with structural color.^[^ ^7^ ^]^ Chemical encoding schemes have been demonstrated at higher bits per pixel, but typically with off‐platform analytical procedures.^[^ ^16^ ^]^ We demonstrate read and write with a single platform of high information density pixels through whole word encoding schemes with autonomous error correction. This scheme is comparable in information storage density to the third IBM drive 1301 (1962) and higher than comparative information densities achieved in multiwell plate chemical encoding schemes.

LiL prints present opportunities to increase information density via two engineering routes: compositional enrichment or further physical miniaturization of droplet pixels. Compositional enrichment may be achieved by adding more pressure lines to the printhead, repeated dried and dissolution runs or in the absence of surfactant, new volumes directly added to or removed from pixels. For storage or synthesis applications,^[^ ^45^ ^]^ additional rounds of complex solutions may be added to indexed spots. Information density may therefore be increased through printing DNA data storage units,^[^ ^44^ ^]^ nanoparticles, molecular libraries or beads. DNA components may enable massive information density.^[^ ^46^ ^]^ Moreover, information encoded in LiL prints offers unique cryptographic opportunities such as encryption and decryption through pixel liquid‐to‐solid phase transitions. Small disturbances in the spatial patterning of the pixel compositions, such as through pixel fusion or compositional diffusion will result in permanent information deletion. Without a similar liquid handling technique, the information is practically impossible to retrieve. Without a guide on the contents of each droplet pixel and therefore how to analyze the LiL print, the information cannot be extracted. Such constraints serve as protective barriers in an information security context. Information within LiL prints can be stored in the solid or liquid states. Our results evidence appreciable stability of crystals under inert conditions, suggesting an alternative medium for long‐term information storage. In addition to non‐invasive, in situ readouts, pixels may be analyzed downstream via matrix‐assisted laser desorption/ionization (MALDI) which has been extensively demonstrated in microdroplet arrays.^[^ ^18^, ^47^, ^48^, ^49^ ^]^

By preserving the liquid state, initially out‐of‐equilibrium biochemical reactions could occur within the LiL print. Information rendered in this context thereby contains 2 spatial, a number of component dimensions and 1 temporal dimension (Table S6, Supporting Information). This broadly establishes a fertile ground for future translation of software principles through repurposing the characteristics of any reaction which produces a readily analyzable product. For example, with signal modulated by bacteria, the information legibility may be tuned by any bacterial, metabolically active chemical component. In the future, translating if/else logic or NAND gates can form the basis of responsive sensors or information release strategies based on preserved, biochemical reactions with engineered strains.^[^ ^50^ ^]^

## 4 Conclusion

Beyond the encoding and storage of information, our method opens avenues in many directions. In principle, the LiL print concept can be directly leveraged for functionalizing surfaces with complex patterns for applications in drug screening and analytics.^[^ ^18^, ^19^, ^51^, ^52^ ^]^ Additional work, however, is needed to facilitate industrially relevant, scalable technologies. Our current printing machinery demonstrates a tradeoff between spatial and compositional printing accuracy with the print time applicable in the context of long‐term information storage or legibility applications. Information recovery requires an analytical method subject to a limit of detection and a potentially finite number of read and write cycles. Moreover, the pixel droplet volume and pitch may be further reduced to increase the liquid patterning density. Maintaining the liquid state requires an oil layer encapsulated and sealed in a compact chamber. The potential of this liquid handling method will be exploited in the future through droplet interconnectivity and crosstalk via surfactant stabilization or hydrogels. Such precise compositional variance is entirely not possible with current inkjet methods for the deposition of fixed liquid compositions into surfactant laden oil. Liquid‐liquid interfaces between droplet pixels, such as droplet interface bilayers will underpin the design of liquid devices and screening assays with highly spatially defined initial distributions.^[^ ^30^, ^40^ ^]^ LiL prints thus enable a group of novel biotechnologies and offer a path to increase the information density and portability of chemical encoding schemes.

## 5 Experimental Section

### Reagents

Fluorescein, sulforhodamine B, dextran Alexa Fluor 405, dextran Alexa Fluor 488, dextran Alexa Fluor 647, trichloro(1H,1H,2H,2H‐perfluorooctyl) silane, chloramphenicol, kanamycin, MHB II powder and PBS 1X were bought from Merck, Germany. PDMS was purchased from Sylgard 184 PDMS Elastomer, DOW. SU‐8 3050 and mr‐dev 600 was purchased from MicroChem Corp. HFE‐7500 oil was purchased from 3M, USA.

### Fabrication of the Droplet Printhead

First, the microfluidic structures of the printhead (5 inlet channels and one outlet channel) were transferred onto a structured Si‐wafer via photolithography. This was used as a master mold for a PDMS imprint. Briefly, a SU‐8 3050 photoresist layer was spin‐coated at 3000 RPM to obtain a height of 50 µm and developed in mr‐dev 600 solution. Afterward, the wafer was treated with trichloro(1H,1H,2H,2H‐perfluorooctyl) silane to prevent sticking of PDMS. PDMS was prepared according to the manufacturer's recommendation and poured onto the wafer. After curing at 80 °C for 1 h, the PDMS layer was peeled off and plasma bonded to a plain PDMS slab. The outlet channel was cut open and shaped into the form of a pyramid with a surgical scalpel under a stereoscope. The channels on the other side, that serve as the inlets were cut open and 30 cm long fused silica capillaries with an inner diameter of 50 and outer diameter of 150 µm were pressed laterally into the channel inlets. The chip, along with the five capillaries, was then attached to a 3D‐printed support using two‐component epoxy glue.

### Experimental Setup

The hydrophobic/hydrophilic microarray printing surface was prepared in a cleanroom according to a previous protocol.^[^ ^18^ ^]^ The plate was placed inside an oil‐tight tray on a xy‐stage (M‐404.6PD and M‐414.1PD, Physik Instrumente, Germany) that was mounted on an inverted microscope (Olympus IX70, Japan). Reagents were stored in Eppendorf tubes and transferred onto the plate via the droplet printhead using five pressure‐driven controllers (Flow EZ, Fluigent, France) ensuring fast changes of the fluid flow. The outlet of the droplet printhead was fixed in a frame and positioned above the plate. A force sensor (FUTEK LSB200, USA) was attached to the print head for calibration. The entire unit with the Eppendorf tubes, printhead and printhead mount was connected to a z‐stage (M‐403.2PD, Physik Instrumente, Germany). Before droplet generation, the plate was calibrated to align the outlet with the array positions and to maintain distance between the plate and nozzle precisely across the entire plate. First, the xyz‐coordinates of three corner points of the array were saved in a custom program to yield a planar approximation of the dimensions of the microarray printing surface. Then, six more points on the surface were calibrated yielding a polynomial surface which accounted for slight deformations in the printing surface. Once the calibration was completed, user‐defined parameters (distance, pressure, speed, xy‐offset, rows‐from‐to) could be added to generate the desired array in a fully automated manner. Our custom program is written in Python and interconnected cameras (CS165CU Zelux, Thorlabs, USA and UK‐1117, ABS GmbH, Germany), the motorized stages and the pressure regulators. Before droplet formation, the printing surface was covered with 3 mL of HFE‐7500 oil. The fluorescent dyes fluorescein (GFP channel), sulforhodamine B (mCherry channel), Dextran Alexa 405 (DAPI channel) and Dextran Alexa 647 (Cy5 channel) were dissolved in PBS to concentrations of 35, 40, 36, and 40 µM respectively. Solutions were transferred to 1.5 mL Eppendorf tubes prior to printing. Comparative dye concentrations were used in other experiments.

### Bacteria Cultivation

Bacterial strains were preserved as cryo‐stocks with 25% glycerol at −80 °C. Before the experiment, 100 µL of an overnight culture was inoculated into 3 mL of cation adjusted MHB II, supplemented with 50 µg mL^−1^ kanamycin sulfate as needed for plasmid maintenance for the ATCC25922 (pSEVA271_sfGFP) strain. The cultures were incubated at 37 °C in a shaking incubator (Minitron, Infors HT) at 200 rpm. Once the OD600 reached 2–4, the cultures were diluted to an OD600 of 0.05 and further diluted in the printhead to a final OD600 of 0.025. Bacteria in the 0‐bit composition were incubated for 1 h in 40 mg L^−1^ chloramphenicol prior to printing. The bacteria underwent 3 pellet and fresh media wash steps at 3000 RPM for 3 min. For the bacterial growth test (0‐bit) chloramphenicol was diluted to a concentration of 1 µg mL^−1^ in cation‐adjusted MHB II supplemented with 50 µg mL^−1^ kanamycin sulfate. This solution was further diluted in the printhead to 0.5 µg mL^−1^. The 1‐bit state was an otherwise comparative sample in the absence of chloramphenicol.

### Fluorescence Imaging and Analysis

For fluorescence measurements, the oil‐tight tray including the generated microdroplet array was transferred to a Ti2 Eclipse (Nikon, Japan) with a SOLA SE II (Lumencor, USA) light source and a DIQ2 camera (Nikon, Japan). The objective used for all measurements was a CFI Plan Apochromat Lambda 10x (Nikon, Japan). The intensity and exposure time was adjusted based on the fluorescence channel: GFP (100 ms and 20%), DAPI (500 ms and 20%), mCherry (150 ms and 20%) and Cy5 (150 ms and 20%). For the bacterial experiments, and all long‐term liquid state experiments the oil tray was sealed with a breathable PCR foil to avoid liquid evaporation during incubation, storage and imaging. Additionally, two small water trays were placed into the oil bath to keep the environment humid. The exposure time of the GFP channel was reduced to 100 ms and 10% intensity and the array plate was imaged every 20 min. We used filter sets for GFP, mCherry, DAPI and Cy5 from Nikon. Image analysis was performed using a custom‐built MATLAB script by extracting the mean fluorescence intensity values of the spots. All microscopy data for LiL prints were stitched together using NIS elements software. In all cases, we used minimal background correction or brightness modifications to the images, adjusting to capture the full dynamic range of each component. Further experimental and analytical detail is supplied in the Supporting Information.

## Conflict of Interest

The method “micro droplet array by stream shearing, MASS” is part of a patent that was submitted on September 17, 2024, under no. EP24200716.9.

## Author Contributions

M.B., R.S., and P.S.D. designed the study. M.B. developed the technology. M.B., R.S., and L.F. developed the method, planned and performed the experiments, processed and analyzed the experimental data and created the figures. C.L.D. and S.B. provided the python script for generating the pressure maps. P.S.D. supervised the study. R.S., wrote the manuscript. M.B., L.F., and P.S.D. reviewed and provided edits that all authors approved. M.B., R.S., and L.F. are co‐first authors.

## Supporting information



Supplementary Information

Supplemental Video1

Supplemental Video2

## Data Availability

The data that support the findings of this study are available from the corresponding author upon reasonable request.
