# Supplementary material for: Liquid‐in‐Liquid Prints: High‐Density Biochemically Encoded Information Preserved in Microdroplet Arrays
Source: Adv Mater. 2025 Nov 24;38(7):e16338. doi: 10.1002/adma.202516338 (PMC12862683; doi:10.1002/adma.202516338)
Supplement: Supplementary file 1 — Supplementary Information [file ADMA-38-e16338-s003.pdf]

ADVANCED MATERIALS

Supporting Information

for *Adv. Mater.*, DOI 10.1002/adma.202516338

Liquid-in-Liquid Prints: High-Density Biochemically Encoded Information Preserved in Microdroplet Arrays

*Maximilian Breitfeld, Robert Strutt, Leonard Fröhlich, Claudius L. Dietsche, Sebastian Bargfrede and Petra S. Dittrich**

Supplementary information: Liquid-in-Liquid Prints: High-Density Biochemically Encoded Information Preserved in Microdroplet Arrays

Maximilian Breitfeld*, Robert Strutt*, Leonard Fröhlich*, Claudius L. Dietsche, Sebastian Bargfrede and Petra S. Dittrich**

Department of Biosystems Science and Engineering, ETH Zürich, Schanzenstrasse 44, Basel 4056, Switzerland

* Co-first authorship
**Corresponding author and e-mail: Petra S. Dittrich, petra.dittrich@bsse.ethz.ch

Contents **Page**

Contents	Page
Supplementary materials and methods	2 - 4
Biochemical structure of printed components	4
Error calculations	5
Positional drift correction	6
Data tables and summary	7-8
Machine overview and summary	9
Print head fabrication	10
Print modes	11
LiL print gallery	12
MASS vs SOS spatial and compositional variance analysis	13 – 15
Droplet cross talk assessment	16
Scannable QR codes	17
ASCII encoding analysis	18
Error spatial distribution	19
Error correction protocol for word encoding	20 – 21
Tree of life scan and pressure map layers	22
References	22

Supplementary materials and methods

The following contains additional notes, methods and details on the printing of specific LiL prints. Across our experiments we varied between 2, 4 and 5 printhead component lines, most prints featuring all 5 lines. In all experiments, 1 line contained the appropriate buffer solution. The buffer itself was varied between experiments. For each pixel, the relative pressure applied to the component lines was determined according to the pressure map. The sum pressure was kept constant through the buffer line per deposition. The absolute pressure applied to all lines was controlled and optimized for printing in both MASS and SOS modes. Our software also allowed control over multiple printing parameters including the printhead offset from the printing surface, the printhead speed, the escape distance from the spot in z and the print delay at the spot amongst other settings[1]. During a print these settings were kept constant and generally constant between prints which we calibrated per experiment. A non-eluting, constant back flux pressure of >2% was applied to channels not included in a deposition. We demonstrate LiL prints at 56 dpi for all examples. To prevent evaporation, we used a sealing film over the printing surface holder (96 well plate dimensions). The liquid patterning of the LiL print was maintained even if the sealed chamber was moved or rotated by hand or automatically shaken.

Image - Mona Lisa – Black and white – MASS and SOS - For the pressure map we converted the face of Leonardo da Vinci's Mona Lisa to black and white and adjusted the resolution. We made minor manual edits to the initial digital processing to improve visualization. This image was selected to test the printing accuracy and precision of different print modes. A black droplet pixel contained no chemical components, only DI water. A white pixel contained 3 components, the denoted RGB fluorescent dyes (Alexa Fluor 647, fluorescein and Alexa Fluor 405 respectively). We printed the image 4 times, with different print modes, speeds and settings (Figure 2 a). Additionally, we printed the images multiple other times for reproducibility. Optimized mass settings were fixed at the stage speed of 1 mm s^{-1} used throughout for other MASS printed examples.

Image – Color Wheel – Color – MASS and SOS - For the pressure map we digitally rendered a color wheel and adjusted the resolution. This image was selected to test the compositional printing precision in terms of switches to components on the fly with MASS printing. MASS resulted in a slightly reduced pixel volume in this experiment which can be seen in comparison between Figure 2 e and f.

Image - Mona Lisa / A Sunday Afternoon on the Island of La Grande Jatte – Color – MASS - The digital images were adjusted in resolution and split into RGB additive channels. Color brightness was correlated to the dilution factor of the PBS buffer line. We denote this color model as a red green blue water (RGBW) color model. We printed with optimized MASS settings as inferred from the black and white experiments.

QR codes (L = 2) – SOS - A 4-layer pressure map was generated where each layer comprised a digital QR code where the width and height was equal to the respective x and y number of droplet pixels (25 * 25). A low or high concentration of a chemical component was assigned to each respective black (0) or white (1) digital pixel. The composite color was here represented by RGB additive, with an additional orange channel layered on top. Without sealage, the surface dried within about 30 minutes due to oil evaporation. In preliminary experiments, we printed the 0-bit as the absence, or 0 concentration of a component. These early versions of the print had similar properties in the liquid state, however, the dried state was not encrypted and could still be resolved since the presence of the component inferred either a 0 or a 1 in each fluorescence channel. After drying, not all components resuspended equally; fluorescein returned to around 70 % of the comparative intensity level potentially due to surface adsorption and incomplete solvation. We kept the resuspended QR codes within a sealed chamber for 3 days, over which no discernible evaporation was observed.

Characters (L = 4) – SOS - We used a simple python algorithm to convert text to a byte and then to a pressure map. We used the full Windows extended ASCII encoding. Each text character was aligned into a block of a given 2D shape. Component concentrations were assigned to a linear combination of 2 bits via a look up table. Since L=4, this covered all bit combinations (00,11,01,10). Each level of the look up table was assigned an exponentially distributed pressure value. We used 'helloworld' as a canonical example message with numerous repetitions of the same compositional identities within the coding block. A baseline concentration of each component was present in every droplet at level 1 which facilitated fluorescence quenching encryption when dried. The canonical string demonstrated bit pairs with bit pair redundancy (DAPI), and both 2 (Cy5) and 4 (mCherry and GFP) fluorescence intensity levels. We assessed the dried and resuspended states using a similar thresholding analysis as in the initial print. We used SOS to print de-ionized water on the crystals

Characters (L = 4) – MASS – To test numerous information accessibility cycles, we created a repetitious 'helloworld' block with MASS, printing each letter column wise on the surface. We used the same components as in prior experiments. Here, we optimized the protocol by redissolving for at least 30 minutes under 37 °C before imaging. As before, we redissolved using SOS printing. We performed the first 4 cycles within an afternoon. Then, for long-term storage, the dried surface was placed in a falcon tube, flushed with nitrogen and placed in a -20 °C freezer. After 7-days, the surface was redissolved and the information assessed. In this experiment, we additionally redissolved a new zone.

Words (L = 6 or 8) – SOS – We used higher L states to investigate our chemical printing precision and to investigate the extremes of our printing method. As above for the byte encoding, we assigned a pressure map according to L = 6 or 8 giving a number of definable droplet compositional identities. To convert whole texts to words, we first extracted all unique words from an original text. Punctuation was removed from the text. We used Lewis Carroll's 'Alice's Adventures in Wonderland' as an example which according to our pipeline contains 2648 unique words. Any whole text with less words than the number of defined droplet compositional states may in theory be encoded. Considering 4096 possible states (8^4) this gave a look up table encoding sparsity of 35% which we initially kept consistent between L = 6 and 8 experiments for comparison. A fixed sparsity of 35% and an L=6 was sufficient to encode the first 40000 words of 'Alice in Wonderland'. Words from the list were randomly assigned to composition identities. We printed the first 100 words of the text alongside L = 4 parity droplets which featured an encoding based on the numerical index of Windows extended ASCII encoding. We printed 7 repeats of the word encoding block on the printing surface. In both experiments, we noticed that errors were not uniformly distributed across components, tending to accumulate in a single component. In processing, we assumed that this was not known, and therefore searched the full space occupied by +-1 errors in the look up table. With additional information on the writing precision per component, error correction may be improved. We additionally tested a scenario of solving the L=6 encoding entirely by hand using printouts of the look up table and the initial word assignments. In this experiment, using human intuition, we could easily recover 100% of the information. Information density in this LiI print was determined by assuming each word character as a byte (as in ASCII encoding). Words were printed in a spatial dimension of 4.5 mm*4.5 mm for the encoding block, and 5 mm*5 mm when including the error correction block.

Programme - Tree of life – Single stage release – MASS – To create the pressure map, we drew a dormant tree of life motif first by hand, which we then scanned into a computer. We made minor edits to the color of the scan and converted it into a color pressure map as above for the Mona Lisa color print. We assigned a fourth channel to the color pressure map, denoting the location of the bacterial culture component. This component grew into the 'leaves' of the tree. We modified stock concentrations to ensure that the relative peak signal from the sfGFP bacteria, was higher than the fluorescein maxima. The bacteria were diluted in the printhead to give an initial approximal droplet OD_{600} of 0.0125.

Programme - Tree of life – Multiple stage release – MASS – We made a grey scale image of the dormant tree scan. We made an additional edit to the scan by adding the location of 'apples' which we assigned a new bacterial culture component. Greyscale was represented by Alexa Fluor™ 405. The other 4 lines were MHB and the two engineered bacterial strains. The sfGFP expressing bacteria were diluted in the printhead to give an initial approximal droplet OD_{600} of 0.0166. The mRuby2 expressing bacteria were diluted in the printhead to give an initial approximal droplet OD_{600} of 0.00325. Both tree of life motifs were printed on the same printing surface and sealed together and imaged overnight. The next afternoon, the seal was removed, the oil evaporated and the dried cultures were imaged using a handheld UV lamp (Figure 1 c).

Programme - QR Code – Release and decay – SOS – We used the former QR code pressure map layer and created a custom pressure map assigning 3 components - two different bacterial cultures and buffer. The initial OD_{600} of 0.025 was kept constant for both cultures. Following printing, the LiL print was immediately sealed and transferred to the imaging microscope. The legibility window width could be extended through intensive additional signal processing although not beyond the 14 h timepoint.

Supplementary table 1: Biochemical structure of printed components. *denotes polysaccharide covalent bond. Alexa Fluor 647 structure from the following reference[2].

		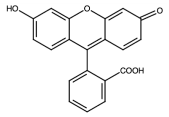	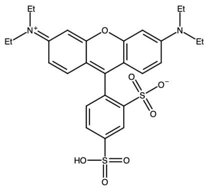	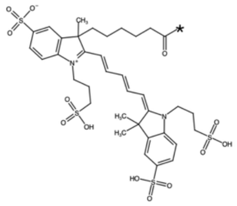	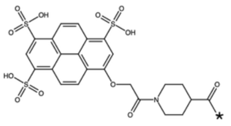
Name:	PBS / Water / MHB	Fluorescein / Alexa Fluor 488	Sulforhodamine B	Alexa Fluor™ 647	Alexa Fluor™ 405
MW (Da):	Buffer / Solvent / Buffer	332.31 / 10000	558.67	10000	10000
Ex/Em peaks (nm):	n/a	498 / 517 490/525	565/586	650/668	401/421
Filter:	n/a	GFP	mCherry	Cy5	DAPI
Color assigned in figures:	n/a	Green	Orange or Red	Red	Blue / Grey
Stock concentration (µM):	n/a	50	50	50	50

	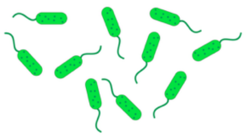	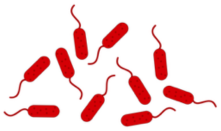
Strain name:	*E.coli* ATCC25922 sfGFP	*E. coli* mRuby2
Expressed protein	sfGFP	mRuby2
Ex/Em peaks (nm):	650/668	559/600
Filter:	GFP	mCherry
Color assigned in figures:	Green	Red
Initial stock OD_{600}:	0.05	0.01

Supplementary note 1 – Error calculations.

Black and white Mona Lisa prints were printed with two calibration droplet columns of black or white pixels alongside. The mean squared error (MSE) was determined via the following equation:

$$MSE = \frac{1}{\#Droplets} \sum_{droplet=1}^{\#Droplets} \sum_{dye=1}^{3} (I_{droplet,dye,normalized} - I_{droplet,dye,mask})^2 \quad (1)$$

Where $I_{droplet,dye,normalised}$ denotes the intensity of a dye component in a droplet and $I_{droplet,dye,mask}$ denotes the corresponding digital value. Data was normalized according to the mean intensity of the calibration droplets between 255 (white) and 0 (black). We investigated two accuracy calculations accounting for two worst case scenario prints setting the maximal error (MSE_{max}).

$$Greyscale\ MSE_{max} = \frac{1}{\#Droplets} \sum_{droplet=1}^{\#Droplets} \sum_{dye=1}^{3} (\frac{255}{2})^2 = 48'768.75 \quad (2)$$

In this case, the lowest accuracy and precision was given by a greyscale between 0 and 255 states.

$$Inverse\ MSE_{max} = \frac{1}{\#Droplets} \sum_{droplet=1}^{\#Droplets} \sum_{dye=1}^{3} (255)^2 = 195'075 \quad (3)$$

In equation 3, each droplet contained the inverse of the intended state and thereby represented minimal accuracy. We denote the above as 'Inverse' and report this in figure 2 for our accuracy calculation as it best accounted for the relative compositional drift in pixel intensities. Precision and accuracy were determined below with the appropriate MSE_{max}.

$$Precision = 100 \times (1 - \frac{MSE}{Greyscale\ MSE_{max}}) \quad (4)$$

$$Accuracy = 100 \times (1 - \frac{MSE}{Inverse\ MSE_{max}}) \quad (5)$$

The above calculation determined the offset value by plotting the MSE as a function of an offset between comparative same position in the LiL print and the digital mask. The binary spatial precision was determined by a threshold-based classification of each $I_{n,}$ in each droplet and compared according to the shift corrected digital mask. In the approximating scenario, a droplet was classified correctly 'black' or 'white' if 2/3 of its $I_{n,}$ values were below or above a threshold of 127.5 repsectively. This was used to determine the percentage of correctly classified droplets.

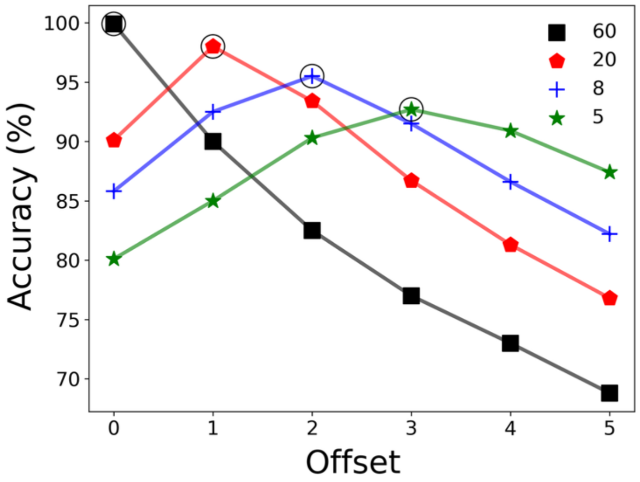

Positional drift correction for MASS printing. Calculated with accuracy – inverse, MSE calculation. Circled data show corrected positional drifts. Leged shows total print times in minutes.

To determine the accuracy in the chemical composition of droplets in the encoding experiments, we used an assessment based on L.

$$\begin{aligned} &Chemical\ printing\ accuracy \\ &= \sum_{droplet=1}^{\#Droplets} \sum_{dye=1}^{4} \left(\frac{\left| Map\ ID_{droplet,dye} - Chemical\ ID_{droplet,dye} \right|}{L} * 100 \right) \end{aligned} \tag{6}$$

Where Map $ID_{droplet,dye}$ is the digital identifier of the dye and the Chemical ID_{dye} is the assigned concentration level of the dye in a printed droplet. The above equation was applied to the L = 6 and 8 conditions, since for L = 2 and 4, we observed no deviations between Map $ID_{droplet,dye}$ and the Chemical $ID_{droplet,dye}$. The information recovery value (Figure 4) informs the number of droplets containing a component with at least one deviation between the Map and Chemical IDs.

Supplementary table 2: Comparison to commercial liquid handling methods – life science applications

Method name	Commercial or academic	Pros	Cons
Inkjet printing	Commercial	- Fast production<br>- pL range deposition<br>- Extreme precision<br>- Non-contact printing	- Deposition only<br>- More complex for liquid-liquid printing<br>- Clogs easily<br>- Fixed volume per deposition<br>- Typically single component per droplet<br>- Mechanical / thermal agitation of cells<br>- Viscosity limited
Acoustic dispensing	Commercial	- Non-contact printing<br>- Varied volume per droplet<br>- No-cross contamination<br>- Fast production	- Deposition only<br>- High cost<br>- Limited liquid compatibility
MASS and SOS droplet printing	Academic	- Preservation of liquid state<br>- Up to 180 hz production*<br>- Total handling (deposition and removal)<br>- Print mode switching<br>- Multicomponent printing<br>- No thermal or mechanical agitation of cells<br>- Varied volume per droplet<br>- Integrated measurement and analysis	- Contact-based printing<br>- Clogging of microfluidic channels<br>- Requirement of patterned printing surface<br>- Speed-accuracy tradeoff in print modes

*under optimized conditions with 50 mm/s stage move speed and reduced spot pitch and diameter.

Supplementary table 3: Printing compositional accuracy and precision for images.

Digital information	Print method	Print time (hr)	Print speed (mm s^{-1})	Precision - Greyscale (uncorrected)	Precision – Greyscale (corrected)	Accuracy – Inverse (uncorrected)	Accuracy – Inverse (corrected)	Binary spatial precision (corrected)
Mona Lisa (B&W)	MASS	0.083	5	20.3%	70.7%	80.1%	92.7%	88.9%
Mona Lisa (B&W)	MASS	0.133	3	43.4 %	81.9%	85.8%	95.5%	94.5%
Mona Lisa (B&W)	MASS	0.33	1	60.4%	92.1%	90.1%	98%	96.8%
Mona Lisa (B&W)	SOS	1	Deterministic	99.5%	99.5%	99.9%	99.9%	100 %
Mona Lisa (Color)	MASS	2	1	n/a	n/a	n/a	n/a	n/a

Supplementary table 4: Encoding schemes

Encoding scheme / Figure	Analytical method	Information density (kB/inch^2) (0.032 inch^2 - 100 droplets)	Approximal information per droplet	Information sum	Number of possible compositions	L	Cn	Write time (hr)	Information recovery – auto correction (%)
QR (bits) / 3	Fluorescence microscopy	1.5	4 bits	400 bits	16	2	4	0.3	100
ASCII (bits) / 3	Fluorescence microscopy	3.1	8 bits	800 bits	256	4	4	0.7	100
Word encoding / 4	Fluorescence microscopy	12.6	8 – 96 bits	414 characters	1296	6	4	1 hr	99
Word encoding / 4	Fluorescence microscopy	12.6	8 – 96 bits	414 characters	4096	8	4	1 hr	87

Supplementary table 5: Encoding schemes in literature

Encoding scheme	Analytical method	Platform	Information density (kB/inch^2)	Approximal information per sample (bits)	Number of possible compositions	L	Cn	Write time	Information recovery (%)	Material state	Reference / year
UGI-bit Images	MALDI	Multiwell	6.47	575	$1.24*10^{173}$	2	575	7.86 hr	97.5	Liquid / solid	Arcadia et al. / 2020.[3]

Inkjet printing fluorescent dyes	Fluorescence microscopy	Glass slide – inkjet deposition	171	8	256	2	8	16 bit/s	99.64	Solid	Nagarkar et al. / 2021.[4]
Mix of complex chemical mixes, machine learning	MALDI	Multiwell	0.079	7	128	2	7	~0.3 hr (12 with drying)	86 (100 with error correction)	Liquid / solid	Gumus et. al. / 2024.[5]
FEAST	^{19}F MRI	Multiwell (and 3D materials)	0.027	22	4.2*10^{6}	2	22	Prepared by hand	Not described	Liquid / gel	Jiang et al. / 2025.[6]

Supplementary table 6: Liquid programmes

Digital information	Print method	Print time (hr)	Print speed	Information legibility	Legibility window
Tree of life	MASS	0.5	1 mm s^{-1}	Single-stage release	Defined by OD
Tree of life	MASS	0.5	1 mm s^{-1}	Two-stage release	Defined by OD
QR code	SOS	0.3	Deterministic	Release and decay	Defined by OD and inhibition

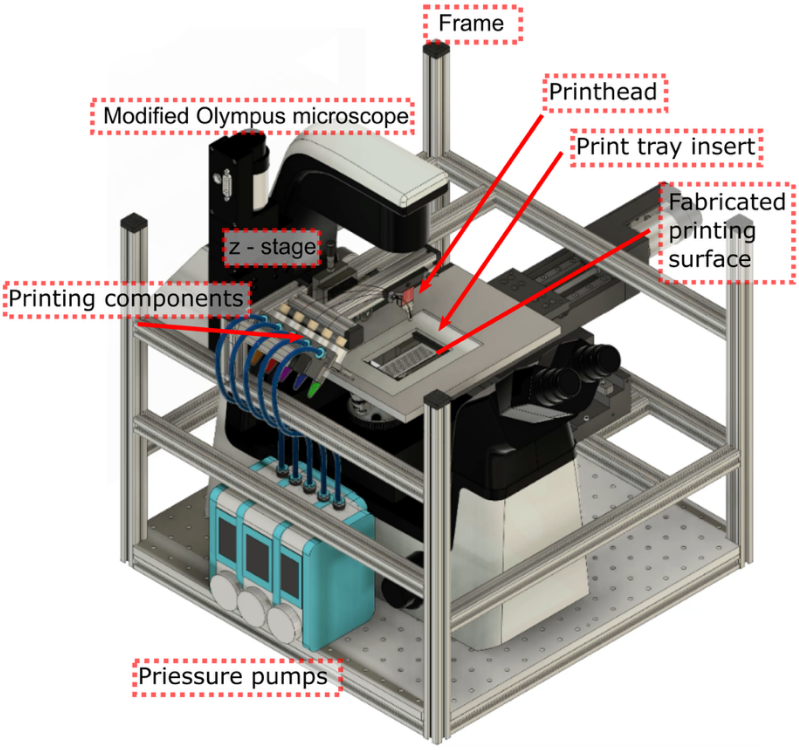

Supplementary figure 1: Printing device and instrumentation. Figure shows CAD render.

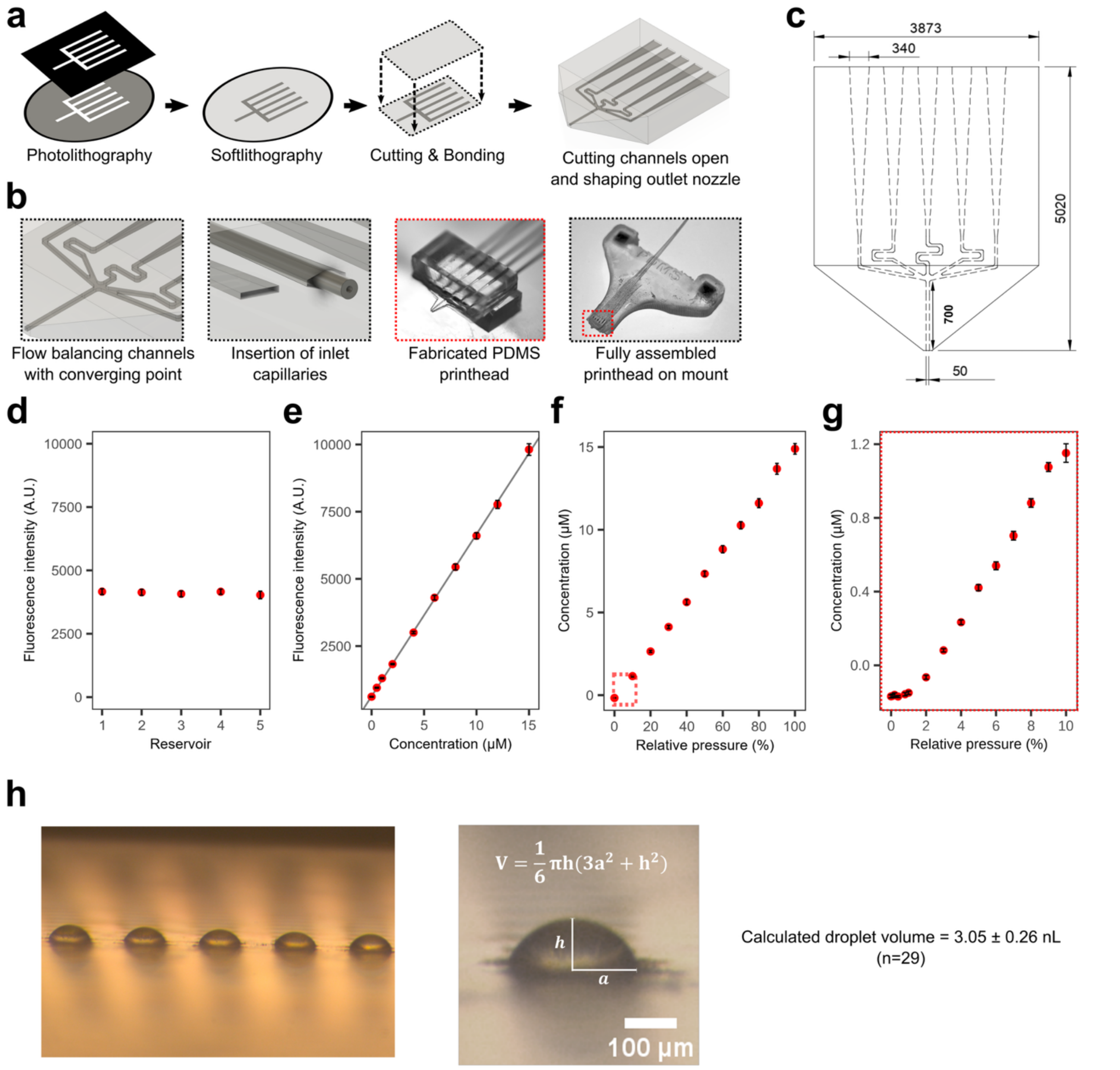

Supplementary figure 2: Printhead fabrication and characterization. **a –** Printhead fabrication overview, following a standard PDMS microfluidic chip production. **b** – Insertion of inlet capillaries into the PDMS piece. The whole piece is attached to a mount which is then attached to a z-stage. Images show CAD and photographs of fabricated assembly. **c** – Dimensions of the PDMS printhead in µm. **d – g.** Characterization of printhead performance. **d –** A measure of the deposition of fluid from each of the 5 inlets on the printhead. Measured as the average fluorescence intensity of 265 droplets printed individually from a single inlet line. Tested per inlet line, from 5 separate reservoirs of 6 µM fluorescein. **e –** The relation between concentration and intensity for fluorescein measured in printed droplets. 10 reservoir solutions between 0 and 15 µM were fed into inlet lines and 53 droplets of each stock concentration were printed and measured. A linear relationship was observed between concentration and fluorescence intensity (R^2 = 0.998). **f** – Fluorescein concentration in printed droplets by mixing two inlet lines. Relative pressure spanning from 0 to 100 % applied to a fluorescein (15 µM) relative to a DI water reservoir. Experiment conducted at a sum, absolute pressure of 1000 mbar. A linear relation was observed between pressure and printed concentration. **g** – Same as in f however spanning 0 – 10 % relative applied pressure. A deviation from linearity was observed <2% relative pressure. This inflection was used to inform the minimal inlet applied pressure to prevent back flow in the printhead and was used for printing throughout. **h –** Characterization of volume determined from a line of printed droplets using a spherical cap equation. Relevant parameters denoted in image, a is the radius of the base cap, h is the height. Characterization photos taken from a low angle using a Dino-Lite camera. Distribution indicates mean and standard deviation of determined volumes. In this figure, all droplets were printed with MASS.

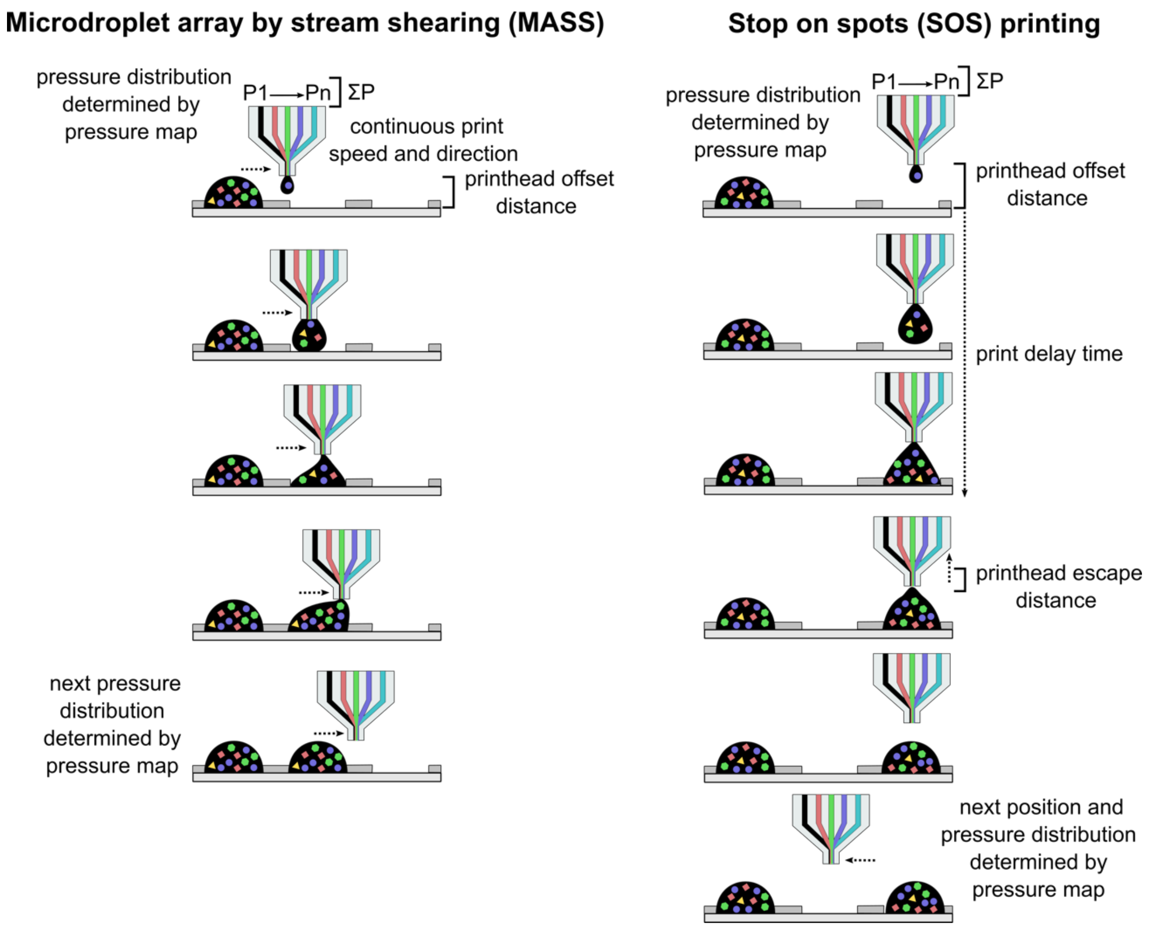

Supplementary figure 3: Different print modes. Cartoons show operational principle of each printing mode. MASS demonstrates on the fly, indexed changes to the droplet composition, SOS relies on deterministic placement of each fixed composition on the print surface.

Supplementary video 1: Droplet generation and resuspension. Videos show the generation of droplets, the drying of droplets through an oil front generating crystals and the addition of water to the crystals, redissolving them.

Supplementary video 2: Dynamic information. Videos show each of the dynamic information motifs presented in Figure 5.

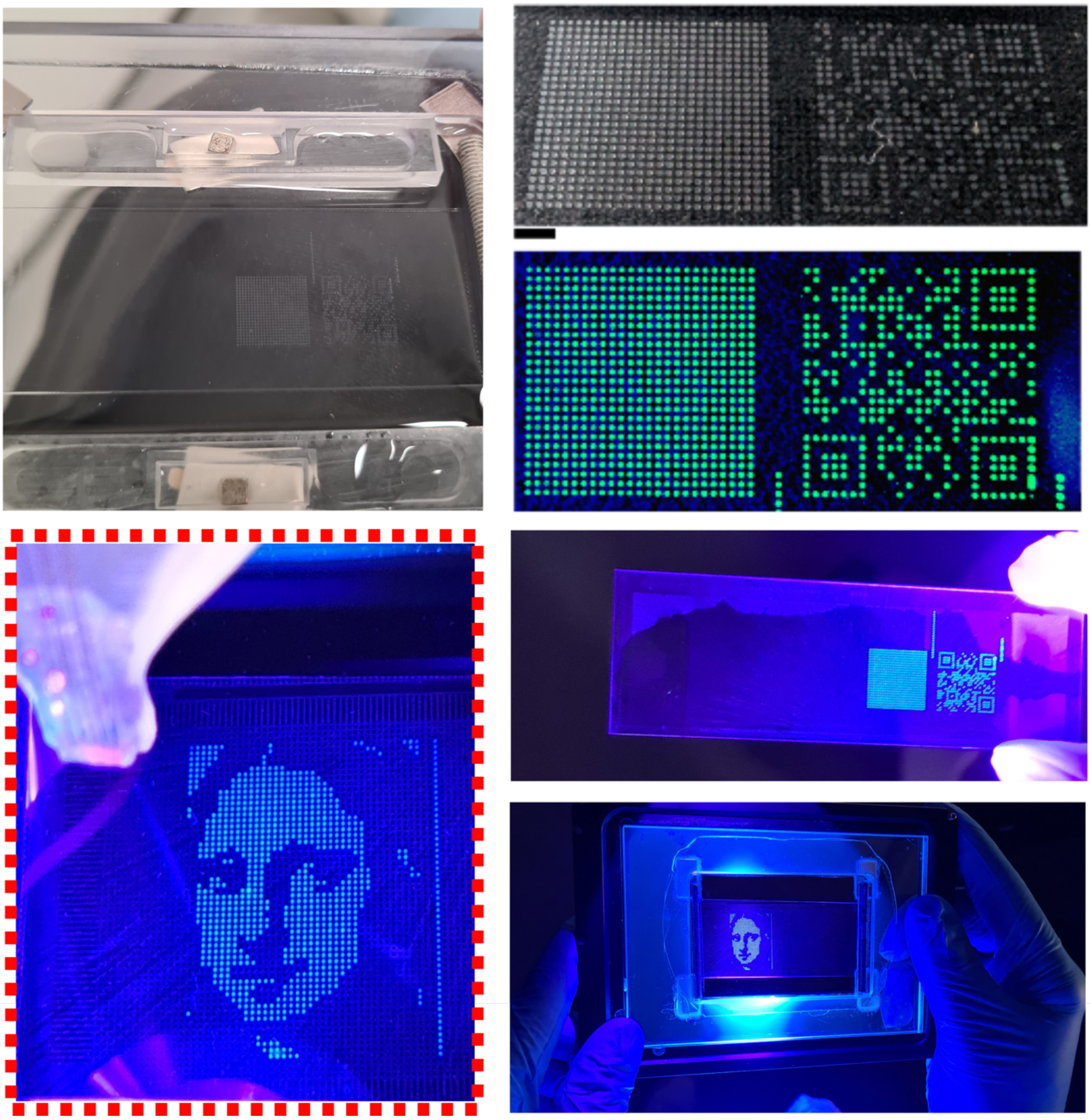

Supplementary figure 4: LiL print gallery. LiL prints under lab condition lighting and when illuminated using UV handheld light sources (green / blue color). The images show LiL prints in various configurations including on the printing stage and handheld in the printing assembly, dried and held as a standalone surface. Scale bar = 2 mm. Note water reservoirs in top left image.

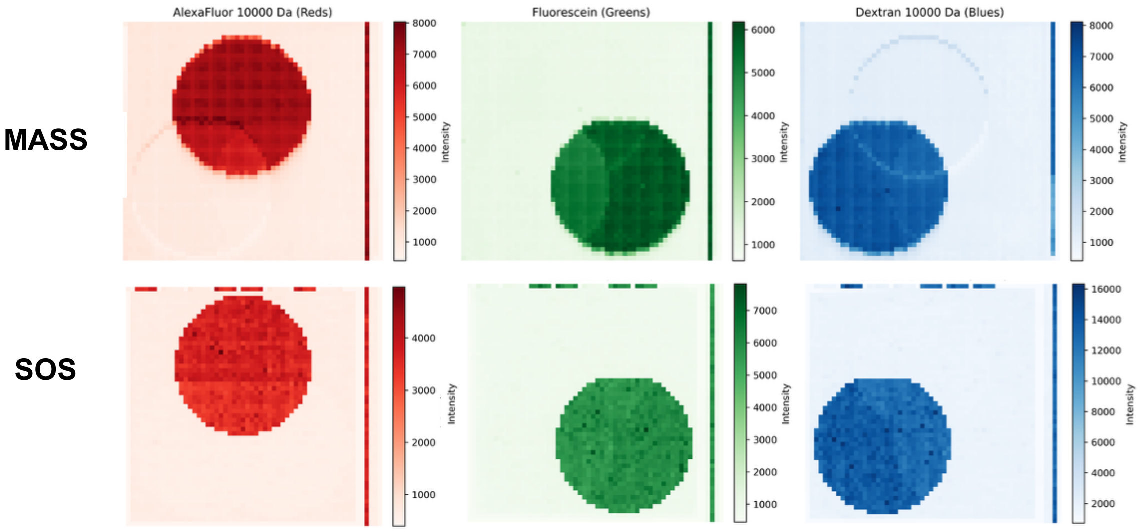

Supplementary figure 5: Spatial distribution of component variance according to print mode. Data show raw distributions from color wheel motif, shown in each channel. Note features present in MASS printing at transitional edges.

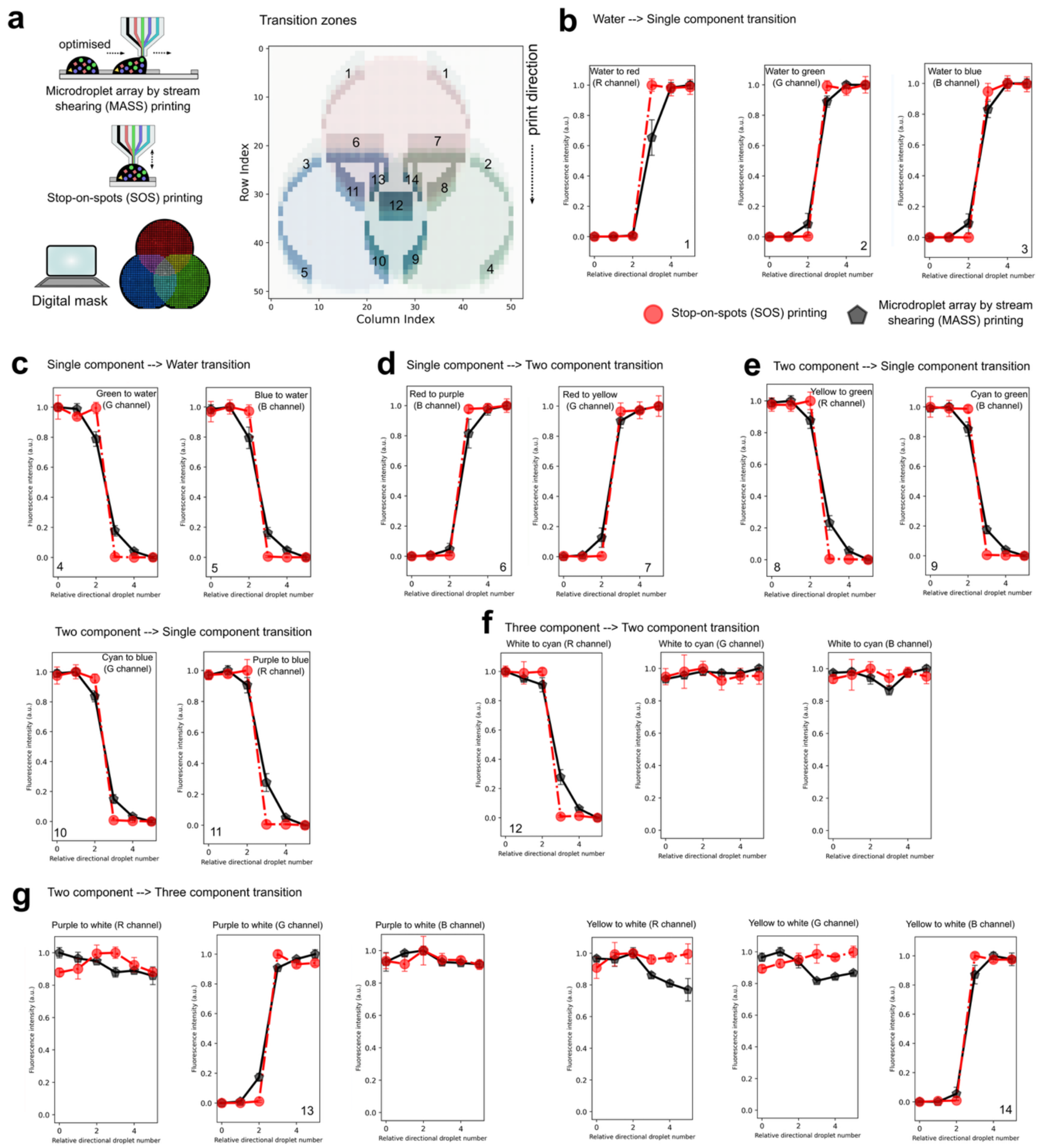

Supplementary figure 6: Transition analysis of color wheel motif. a – Numbered schematic showing analyzed transition regions. Indicated numbers align with sub figures. Each block of 6 droplets was normalized around the center of the transition to align repeat regions per analysis. Each sub figure shows the channels in each transition. For example, a transition from red (only red component) to purple (red and blue components), shows an increase in the blue channel. **b** – Water to single component transitions (numbers 1-3). **c –** Single component to water transitions. Note due to print direction there was no red to water transition in the motif (numbers 4-5). **d** – Single component to two component transitions (numbers 6-7). **e** – Two component to single component transitions (numbers 8-11). **f** – Three component to two component transitions (number 12). **g** – Two component to three component transitions (numbers 13-14).

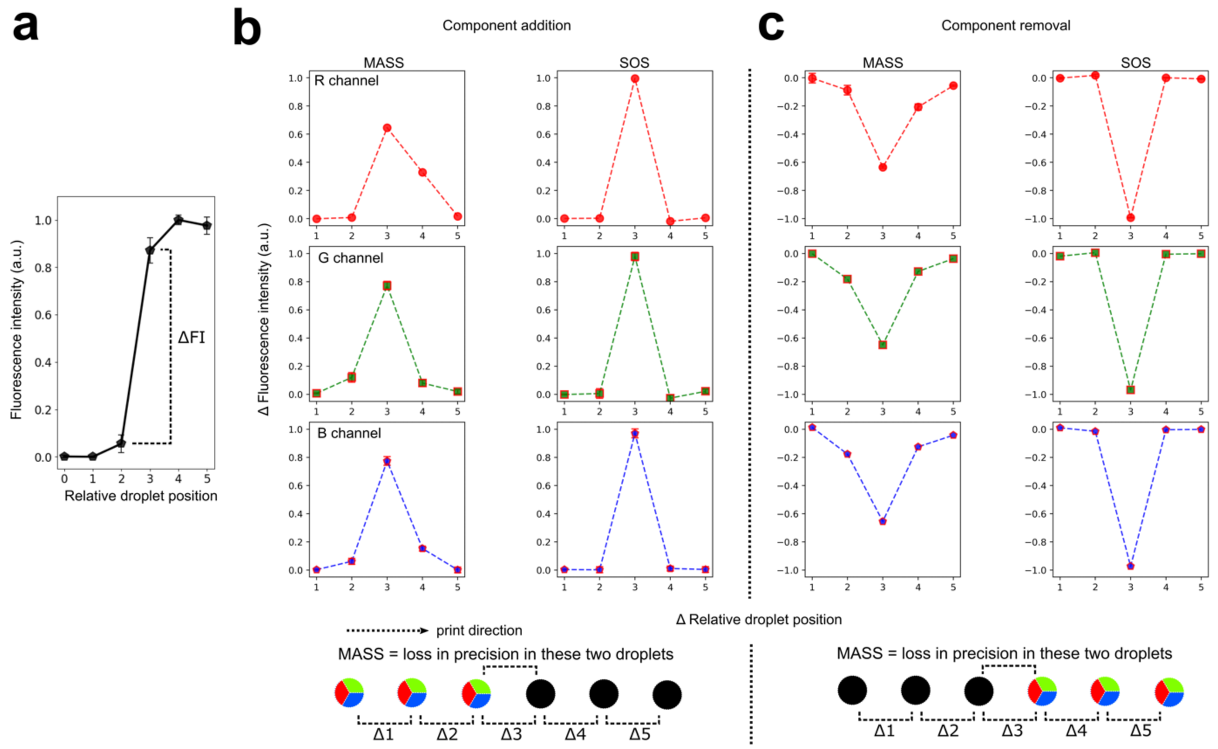

Supplementary figure 7: Mean analysis of color wheel distributions. a – Example of ΔFI calculation. A value close to 1 between droplets 2 and 3 (Δ3) is indicative of a step function in compositional arrangement. All addition or removal transitions of a component are summarized via mean ΔFI. For example, for the addition of fluorescein (G channel) numbers 2 and 7 (Supplementary figure x) were taken. **b** – Analysis of component addition. **c –** Analysis of component removal.

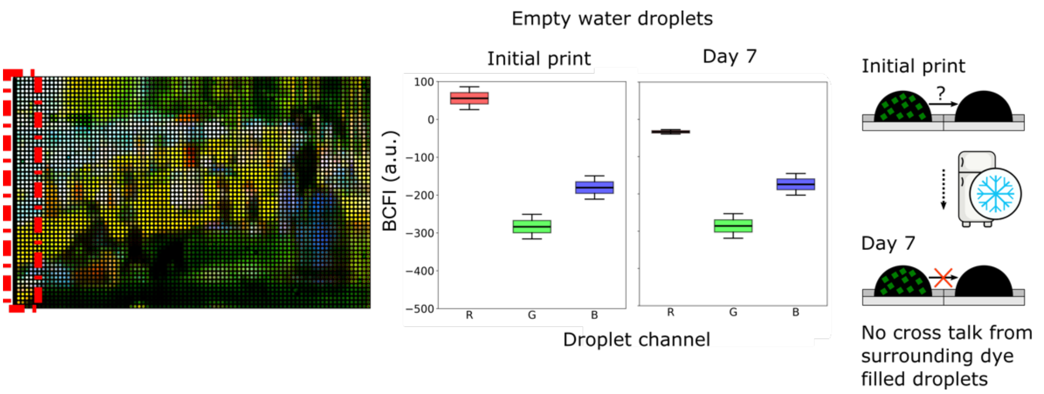

Supplementary figure 8: Droplet cross talk is minimal in LiL prints. Empty, water droplets were printed initially without any of the components that surrounded them. Over the 7-day storage of the LiL print, we observed no deviation in apparent fluorescence intensity in water droplets in green blue or red channels. The slight decay in red signal was due to a deviation between backgrounds. Signal analyzed from 56 water droplets in the annotated region (BCFI: Background corrected fluorescence intensity).

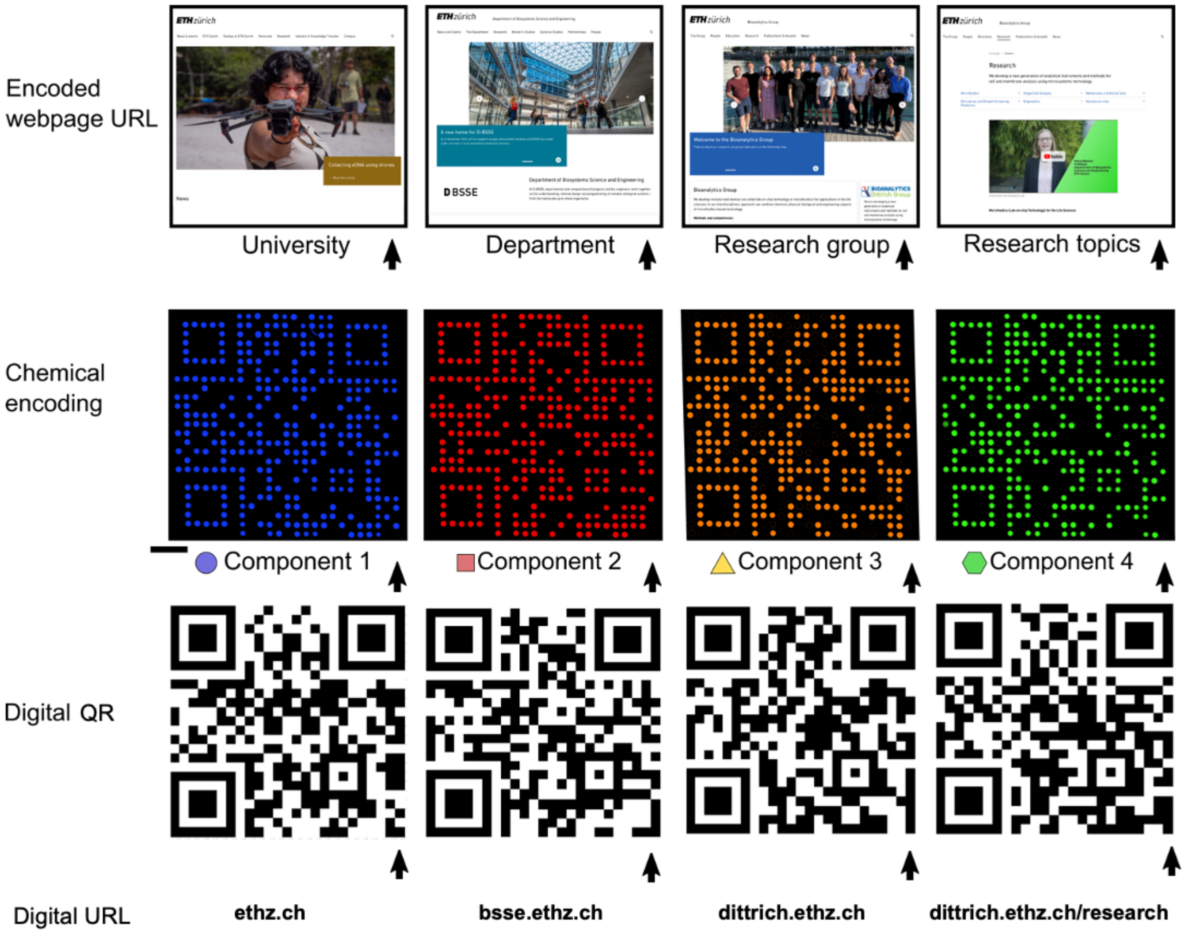

Supplementary figure 9: Scannable QR codes. From bottom to top. The digital URL, is converted to a digital QR code via qrcode 8.2 (https://pypi.org/project/qrcode/), a pressure map was created and printed in a LiL print, such that each component contains a QR. Finally, the recovered website from scanning the QR, screenshot correct from date of experiment.

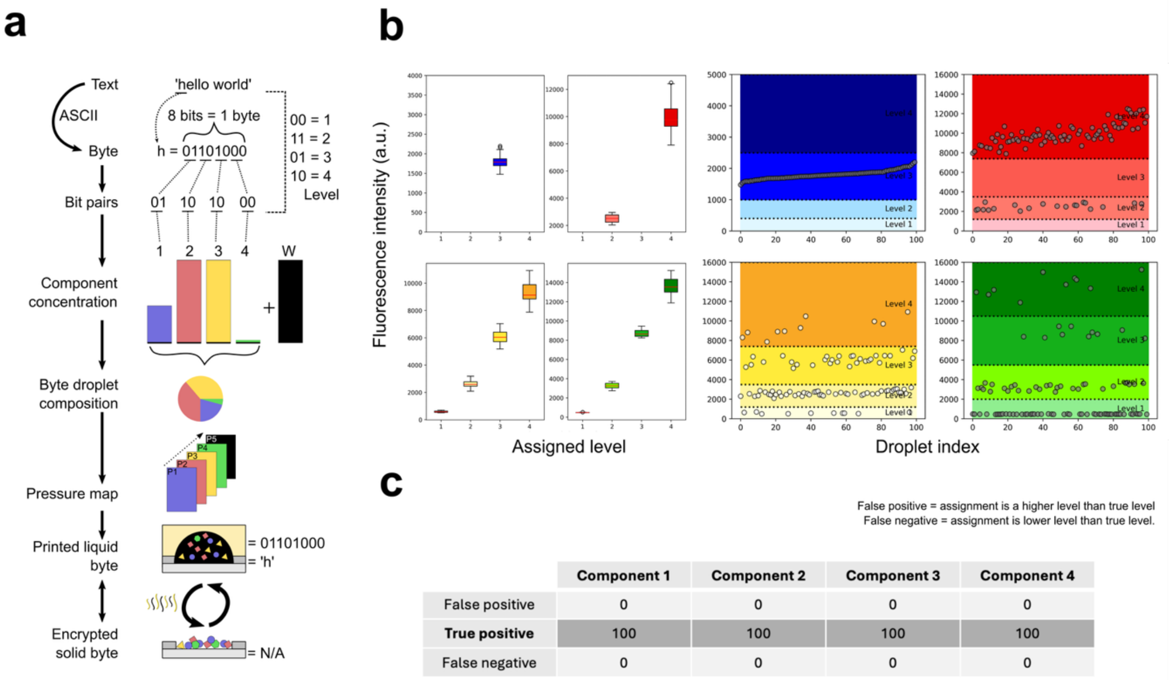

	Component 1	Component 2	Component 3	Component 4
False positive	0	0	0	0
**True positive**	100	100	100	100
False negative	0	0	0	0

Supplementary figure 10: ASCII component level analysis to assign bit pairs. a – Detailed schematic of ASCII encoding process. **b -** Analysis conducted on byte encoded droplets, n = 100. Within a component, populations of the assigned levels were all statistically significant, p-values two tailed t-test <0.001 (***). **c**– Confusion matrix analysis between assigned bit pairs and true bit pairs of the digital code according to analysis in b.

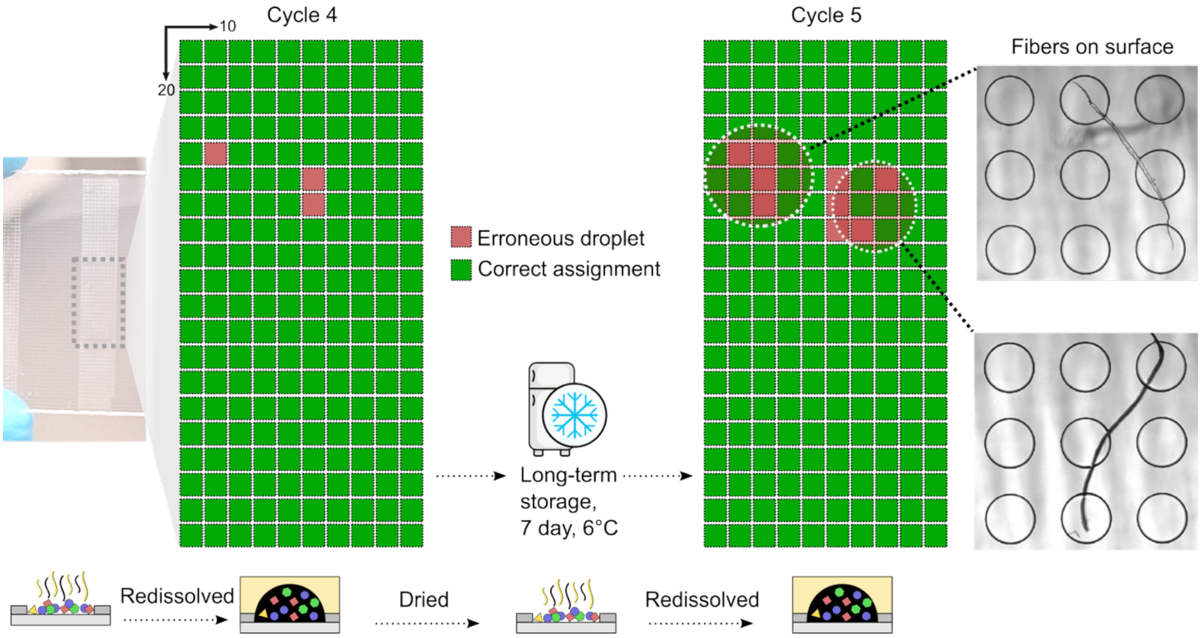

Supplementary figure 11: Error accumulation was spatially constrained during information access cycles due to localized debris. Cartoon showing the distribution of errors on the surface during cycled encryption-decryption runs. Hairs and fibers which became entrapped within droplets were moved during the redissolution step, resulting in slight compositional cross talk, which modulated the fluorescence intensity and thereby the byte string.

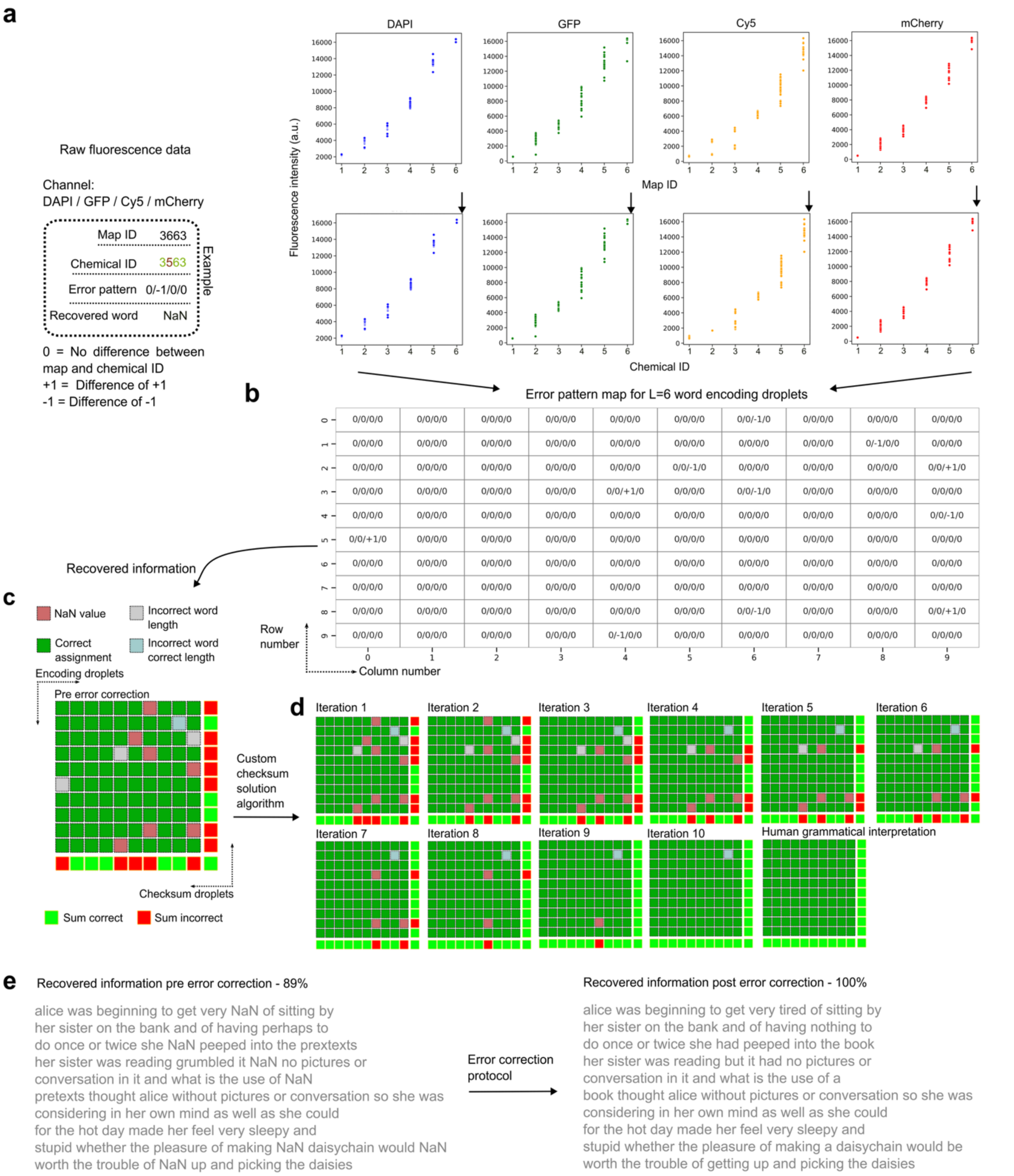

Supplementary figure 12: Error correction protocol results for word encoding. a – Raw data of fluorescence in each channel of word encoding droplets shown against the digital Map ID and the assigned Chemical ID according to thresholding assignment of the data (n=100, L=6). **b –** Error distribution map between map and Chemical ID. **c -** Legend showing color notation for recovered information. Initial grid shows the result pre-error correction, containing 11 erroneous droplets, according to the Chemical ID. We initially reviewed the incorrect word length droplets to assign erroneous positions to the algorithm based on grammatical error. The color in the checksum droplets denotes the identification of errors according to the encoding character sum. **d** – The result of each iteration of the checksum error correction algorithm. The final iteration represents a human review of the grammatical logic of the recovered text. **e**– The recovered information pre and post error correction protocol. The final information is the same as the intended, digital information. The human inferred edit for the incorrect word correct length droplet was a change from ‘nothing’ to ‘perhaps’.

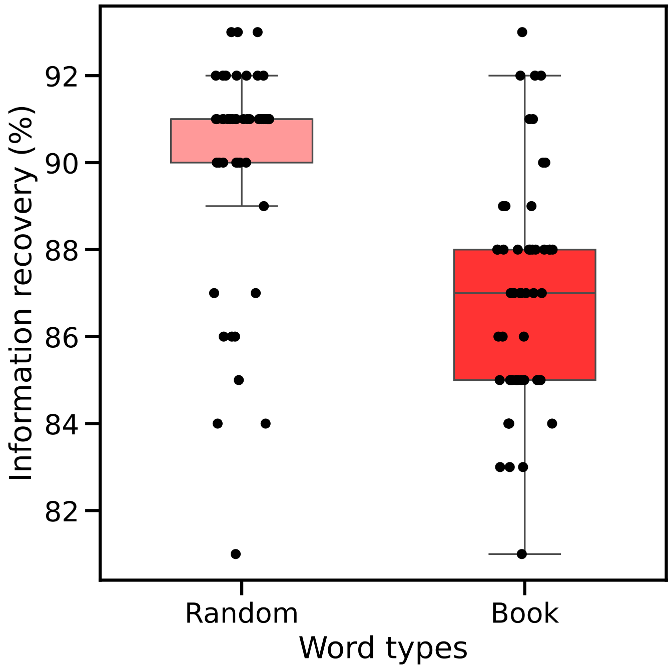

Supplementary figure 13: Lexicon variance. Assessing information recovery with a library of English words and with those from Lewis Carolls' Alice in Wonderland ('Book') for L =8 word encoding scheme. In each case, the error correction algorithm was run 50 times.

Supplementary note 2 – Sparsity calculation

To calculate the encoding sparsity, we used the following equation:

$$Sparsity = 100 - \left(\frac{L^{Cn} - N_{nan}}{L^{Cn}} * 100\right)$$

Where N_{nan} is the number of non-encoding entries in the look up table. The number of encoded words in the look up table was L^{Cn} - N_{nan}.

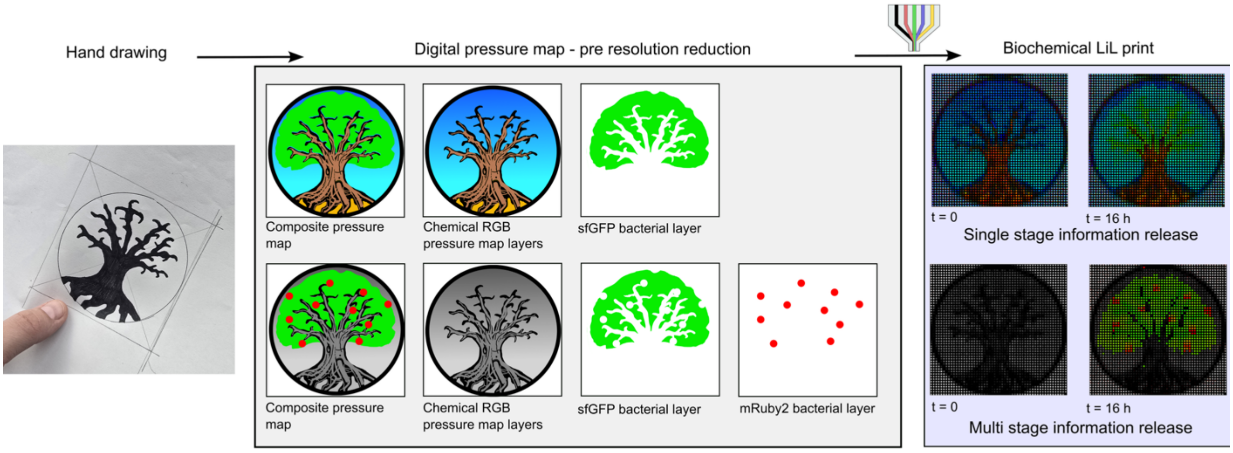

Supplementary figure 14: Design and production protocol for Tree of life motif LiL prints. Note deviation from bacterial pressure map layer is due to slight compositional transition at the zone edges due to MASS printing.

References

1. Breitfeld, M., Dietsche, C. L., Saucedo-Espinosa, M. A., Berlanda, S. F. & Dittrich, P. S. Ultrafast Formation of Microdroplet Arrays with Chemical Gradients for Label-Free Determination of Enzymatic Reaction Kinetics. *Small* 5, 2410275 (2025).
2. Gebhardt, C. *et al.* Molecular and Spectroscopic Characterization of Green and Red Cyanine Fluorophores from the Alexa Fluor and AF Series**. *ChemPhysChem* 22, 1566–1583 (2021).
3. Arcadia, C. E. *et al.* Multicomponent molecular memory. *Nat Commun* 11, 691 (2020).
4. Nagarkar, A. A. *et al.* Storing and Reading Information in Mixtures of Fluorescent Molecules. *ACS Cent Sci* 7, 1728–1735 (2021).
5. Gumus, S. *et al.* Repurposing Waste Chemicals for Sustainable and Durable Molecular Data Storage. *ACS Omega* 9, 19904–19910 (2024).
6. Jiang, Y., Fan, Y., Chen, L., Lin, H. & Gao, J. Super-multiplexed imaging and coding in the range of radio frequency. *Nat Commun* 16, 2567 (2025).
